# Replacement of chromosome 3D with *Thinopyrum* chromosome 3St led to increased drought tolerance during the flowering stage in wheat

**DOI:** 10.1007/s00299-025-03632-5

**Published:** 2025-10-18

**Authors:** Edina Türkösi, Klaudia Kruppa, Éva Darkó, Balázs Varga, Márton György, Zsolt Gulyás, Kristóf Jobbágy, Kateřina Holušová, Eszter Gaál, Balázs Kalapos, Mónika Cséplő, András Farkas, László Ivanizs, Péter Mikó, Andrea Gulyás, Norbert Hidvégi, Péter Kovács, András Cseh, Márta Molnár-Láng, Jan Bartoš, Éva Szakács, István Molnár

**Affiliations:** 1 Centre for Agricultural Research, Hungarian Research Network (HUN-REN), Agricultural Institute, 2462 Martonvásár, Hungary; 2 Institute of Experimental Botany of the Czech Academy of Sciences, Centre of Plant Structural and Functional Genomics, Olomouc, 779 00 Czech Republic

**Keywords:** Agropyron glael, *In situ* hybridization, Genotyping-by-sequencing coverage analysis, Drought tolerance

## Abstract

**Key message:**

The stable 3St(3D) substitution line offers promising genetic potential for improving drought tolerance in wheat during critical reproductive stages.

**Abstract:**

The flowering stage is highly susceptible to drought, which significantly reduces wheat grain yield globally. Low genetic diversity in wheat further limits the discovery of optimal gene variants for breeding climate-resilient varieties. The substitution of chromosome 3D by a group 3 chromosome pair from *Thinopyrum intermedium* × *Th. ponticum* artificial hybrid was identified using *in situ* hybridization and genotyping-by-sequencing. This homoeologous substitution showed good functional compensation for grain yield and fertility, similar to the wheat parents ('Mv9kr1' and 'Mv Karizma') in field and greenhouse trials. The substitution line exhibits a semidwarf phenotype due to the *Rht8* and *Rht2* dwarfing alleles. Automated shoot phenotyping after a 10-day water withdrawal at flowering revealed efficient water preservation allowing to maintain photosynthetic functions, sustained photosynthetic activity, and less chlorophyll degradation, indicated by Normalized Difference Vegetation Index (NDVI) and modified Normalized Difference Index (mND705) values and moderate level of protective functions shown by the expression of stress-related genes. Compared to the wheat parents, the substitution line developed thicker roots with increased volume under drought, resulting in a lower surface-to-volume ratio. This may enhance water storage efficiency and help reduce yield loss under drought conditions.

**Supplementary Information:**

The online version contains supplementary material available at 10.1007/s00299-025-03632-5.

## Introduction

Bread wheat (*Triticum aestivum* L., 2n = 6x = 42, BBAADD) is one of the world's major staple foods, being grown on a wider area than any other crop, with a yield of 784.91 million metric tons in 2024 (FAOSTAT 2024). Wheat now accounts for one-fifth of total food calories and protein intake worldwide (Ceoloni et al. 2017; Erenstein et al. 2022). However, wheat yield is influenced by both biotic factors (fungi, viruses, bacteria, insects, and nematodes) and abiotic challenges (heat, drought, cold, and salinity) that pose threats to global wheat production (Afzal et al. 2015; Kong et al. 2023). Drought is one of the most common climate-related calamities, affecting significant areas of the world and often having disastrous repercussions for agricultural production. In the face of accelerating climate change and reduction of the arable land, breeding efforts must focus on the development of high-yielding, stress-resistant/tolerant crops using innovative breeding approaches to meet the demands of a continuously growing global population (Alam et al. 2024).

Water accounts for 80–95% of the plant’s fresh biomass and plays an important role in numerous physiological processes such as plant growth, development, and metabolism (Wahab et al. 2022). Drought stress occurs when the available soil water becomes depleted, and atmospheric conditions promote continuous water loss through transpiration or evaporation. Even the most prolific agricultural regions face brief times of drought inside practically every year, and occasionally years with severe droughts. Wheat plant susceptibility to abiotic stresses is primarily determined by the duration, frequency, and strength of the stressors (Barnabás et al. 2008; Farooq et al. 2009). Drought causes significant yield loss and hinders wheat performance at all growth stages, although it is particularly severe during the reproductive and grain-filling phases (terminal drought) (Reynolds et al. 2005). The decline of yield is primarily caused by lower photosynthesis rates (Siddique et al. 2000), degradation of chlorophyll and stomatal closure (Cruz de Carvalho 2008; Farooq et al. 2009), increased leaf senescence (Yang et al. 2003), pollen sterility (Dorion et al. 1996), inadequate grain set and development (Ahmadi and Baker 2001; Nawaz et al. 2013), reduced sink capacity (Liang et al. 2001). Crop phenotypic trait measurement in controlled and field conditions has significantly improved over the past 20 years due to the quick development of nondestructive sensing and imaging technology (Jangra et al. 2021; Tao et al. 2022). With a high-throughput phenotyping device, multi-dimensional phenotypes may be accurately acquired and analyzed at various phases of crop growth (Song et al. 2021), both during and after drought stress.

Species related to wheat represent an invaluable source of genetic variability for enriching and diversifying the wheat genome. Recent genomic advances and the availability of reference genome sequences of wild and cultivated relatives of wheat have significantly boosted the efficacy and throughput of chromosomal engineering through the introgression of alien chromatin (Boehm and Cai 2024). *Thinopyrum* (wheatgrass) species are perennial relatives of wheat that have been known as excellent genetic resources in wheat improvement, providing allelic diversity that can mitigate the effect of a wide range of biotic and abiotic stresses (Ali and Mujeeb-Kazi 2020). Several disease resistance genes have been introgressed from wheatgrass species because of their easy crossability with wheat (Li and Wang 2009), such as leaf rust (*Lr*) (Sharma and Knott 1966; McIntosh et al. 1977; Friebe et al. 1992, 1993, 1996), stem rust (*Sr*) (Sears 1973; McIntosh et al. 1977; Kim et al. 1993; Friebe et al. 1996), and yellow rust* (Yr)* (Liu et al. 2013; Huang et al. 2014; Hou et al. 2016; Guo et al. 2023) resistance genes. Wheatgrass species are also well-known for their ability to withstand harsh environmental conditions. Their tolerance to heat (Giovenali et al. 2023), drought (Lu et al. 2022; Kruppa et al. 2025), and salt (King et al. 1997; Tounsi et al. 2024) can also be incorporated into the wheat genome by chromosomal engineering techniques.

From the *Thinopyrum* genus, the hexaploid *Thinopyrum intermedium* (intermediate wheatgrass) (Host) Barkworth & D.R. Dewey (2*n* = 6*x* = 42, StStJ^r^J^r^J^vs^J^vs^) and the decaploid *Thinopyrum ponticum* (tall wheatgrass) (Podp.) Barkworth & D.R. Dewey (2*n* = 10*x* = 70, JJJJJJJ^s^J^s^J^s^J^s^) have been largely used in introgression breeding programs (Li and Wang 2009; Qi et al. 2015; Kruppa and Molnár-Láng 2016; Lang et al. 2018; Jiang et al. 2024). Agropyron glael, an artificial hybrid of *Th. intermedium* (formerly *Agropyron glaucum*) and *Th. ponticum* (previously known as *Agropyron elongatum*), produced by N.V. Tsitsin (Tsitsin 1979), was included in the pre-breeding program in Martonvásár beginning in 2001, to increase wheat genetic diversity through new allelic variants (Molnár-Láng et al. 2012). The chromosomal composition of the wheat × A. glael hybrid, and that of the partial amphiploids resulting from its backcross with the recurrent (wheat) parent were described in detail by Kruppa and Molnár-Láng (2016) and Kruppa et al. (2016, 2025). The first stable introgression lines with desirable agronomic traits were selected in BC_2-3_ generations and described by Türkösi et al. (2024) and Kruppa et al. (2025).

Screening large pre-breeding populations for the presence of alien chromatin is crucial for chromosome-mediated gene transfer into wheat (King et al. 2018). The complex genome and subgenomes of A. glael, harboring chromosomes from *Th. intermedium* (St, J^r^ and J^vs^) and *Th. ponticum* (J and J^s^) and most probably rearranged chromosomes of them were examined using genomic and fluorescence *in situ* hybridization (GISH and FISH) approaches by Kruppa and Molnár-Láng (2016), Kruppa et al. (2016), and Türkösi et al. (2024). However, when investigating closely related genotypes with large genomes, these methodologies were insufficiently accurate, allowing minor alien introgressions (cryptic translocations) or even larger introgressed chromosomal segments to stay undetected (Kruppa et al. 2025). To overcome these limitations, molecular cytogenetic methods are often supplemented with molecular marker analyses to identify *Thinopyrum* chromosomes. Simple sequence repeats (SSR) and Sequence-Tagged Sites (STS) (Ayala-Navarrete et al. 2010), expressed sequence tags (EST) sequences (Wang et al. 2010; Li et al. 2021), PCR-based landmark unique gene (PLUG) markers (Hu et al. 2014; Zhan et al. 2015; Lang et al. 2018) and specific-locus amplifed fragment sequencing (SLAF) markers (Li et al. 2016) have been employed successfully to identify *Thinopyrum* chromosomes in the wheat background. However, low marker coverage of chromosomes continued to limit the complete characterization of *Thinopyrum* genomes, the detection of possible microintrogressions, and the determination of individual wheat-*Thinopyrum* homoeologous relationships. More recently, Cseh et al. (2019) developed and validated a set of 634 chromosome-specific SNP markers for detecting *Th. intermedium* chromosomes or segments in wheat-*Th. intermedium* introgression lines using a new array-based single-nucleotide polymorphism (SNP) marker technique.

The advent of next-generation sequencing technologies has enabled genome-level analysis of wheatgrass species, including the production of chromosome-scale reference sequence assembly of *Th. intermedium* (http://phytozome.jgi.doe.gov/). The final assembly contains 21 chromosomes, encompassing 99.992% of the assembled sequences, with their numbering and orientation determined by the subgenome groups J (cytogenetically designated as J^r^), S (cytogenetically designated St), and V (cytogenetically designated J^vs^). Besides the fact that the production of telomere-to-telomere (T2T) reference sequences for large pre-breeding populations is still not realistic, reference assemblies of wide gene source species significantly facilitated the the high-resolution SNP genotyping of introgression breeding populations using reduced representation sequencing technology (Galla et al. 2018). One of the most cost-effective methods in this field is the double-digest restriction endonuclease sequencing (ddRADseq), a variant of genotyping-by-sequencing (GBS) approaches (Poland et al. 2012a). The main steps of this method are DNA fragmentation using frequent cutter and rare-cutter endonucleases, followed by adapter ligation, size selection, and sequencing by short-read technologies (Wickland et al. 2017). GBS is an excellent tool for genetic mapping (Baird et al. 2008; Elshire et al. 2011; Poland et al. 2012a), breeding applications (Poland et al. 2012b), and diversity investigations (Fu et al. 2012). A quicker and easier-to-use application of GBS sequencing data for the detection of alien introgressions and identification of wheat-alien homoeologous relationships for the introgressed chromosome is based on the mapping of raw reads to the reference genomes of the parental lines, followed by the calculation of relative read coverage values for each wheat and alien chromosomes. This approach has been successfully used to detect introgressed chromatin from *Hordeum vulgare/Hordeum bulbosum* (Keilwagen et al. 2019), rye (Tikhenko et al. 2024), *Thinopyrum* (Adhikari et al. 2022), and also from A.glael (Kruppa et al. 2025) into wheat.

This work reports on the development and extensive investigation of a wheat-A. glael 3St(3D) disomic substitution line that displays semi-dwarfness and a robust drought tolerance exhibited during the heading and flowering stage. The goals of this study were as following: (1) to precisely determine the chromosome composition of the introgression line using genomic and fluorescence *in situ* hybridization and to assess the macrosynteny of introgressed chromosome with wheat through genotyping-by-sequencing (GBS) read coverage analysis; (2) to thoroughly investigate the morphologic and agronomic traits of the substitution line and those of the wheat parental genotypes under field and greenhouse conditions; (3) to assess the impact of drought stress on shoot and root system and yield-determining parameters of the substitution line (4) to investigate biochemical responses to drought stress of the plants carrying the 3St *Thinopyrum* chromosome.

## Material and methods

### Plant material

**Fig. 1:**
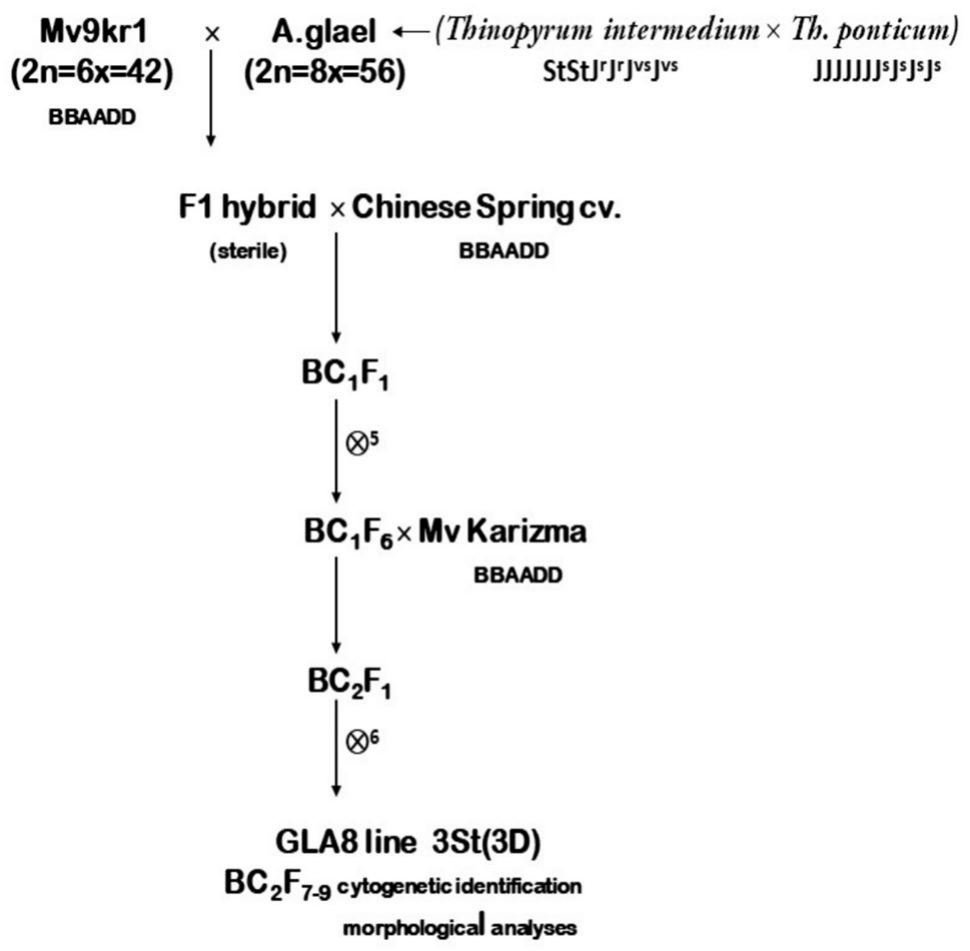
Crossing strategy for the development of GLA8 substitution line. The introgression line was identified in the BC_2_F_7_ generation using molecular cytogenetic methods, and was evaluated morphologically in subsequent generations (⊗ = selfing)

Wheat genotype Mv9kr1 (hereinafter Mv9), which carries the recessive crossability alleles *kr1kr1kr2kr2* (Molnár-Láng et al. 1996), was crossed with A. glael, an artificial hybrid of *Thinopyrum intermedium* and *Th. ponticum*. The sterile F_1_ hybrids were multiplied in tissue culture, and the regenerated plants were crossed with the Chinese Spring wheat cultivar. A subset of BC_1_F_1_ progeny was self-pollinated, and numerous leaf rust and stripe rust resistant partial amphiploids with 56–58 chromosomes were identified and characterized (Kruppa et al. 2016). Another subset of BC_1_ offspring was backcrossed with the facultative Martonvásár elite wheat cultivar Mv Karizma (hereinafter KAR). The lines were maintained in the field for several years while molecular cytogenetic analyses were performed to determine the chromosomal composition of the progeny. In the BC_2_F_7_ generation, a stable disomic substitution line was selected and examined in field trials between 2021 and 2024. Figure 1 shows the crossing strategy for developing the stable, disomic substitution line (designated hereinafter GLA8).

### Molecular cytogenetic investigations

Mitotic chromosome preparations were made from germinating root tips as described by Lukaszewski et al. (2004). The genomic *in situ* hybridization (GISH) approach was used to visualize A. glael (*Thinopyrum*) chromosomes in the wheat genetic background. To detect *Thinopyrum*-derived chromosomes, fluorescently labeled whole genomic DNA from diploid *Th. bessarabicum* (2*n* = 2*x* = 14, J^b^J^b^) labeled with biotin-11-dUTP (Roche, Mannheim, Germany) as J-genomic probe, and DNA from diploid *Pseudoroegneria spicata* (2*n* = 2*x* = 14, StSt) labeled with digoxigenin-11-dUTP (Roche) as St-genomic probe were utilized (Kruppa and Molnár-Láng 2016). The GISH and FISH techniques were carried out as described in detail by Kruppa et al. (2025). To determine the chromosomal composition and hybridization patterns of the wheat/*Thinopyrum* introgression line, GISH and FISH signals were visualized using an AxioImager M2 fluorescence microscope (Zeiss, Oberkochen, Germany) equipped with an AxioCam MRm CCD camera (Zeiss, Oberkochen, Germany) and filter sets appropriate for DAPI (Zeiss Filterset 49), Alexa Fluor 488 (Zeiss Filterset 38), and Rhodamine (Zeiss Filterset 20). The images were processed with AxioVision 4.8.2 software (Zeiss, Oberkochen, Germany).

### GBS library construction, sequencing, and read coverage analysis

Genomic DNA was isolated from fresh young leaves of the parental genotypes Mv9 and KAR, the A. glael, and the GLA8 line in 96-well plates using the BioSprint DNA kit (Qiagen Inc.) according to the manufacturer's instructions. Yang et al.'s (2016) procedure, with minor adjustments described by Kruppa et al. (2025), was used to develop the double-digest restriction site-associated DNA (ddRAD) library. Briefly: The restriction enzymes used were MspI and SphI, the sequences of P1 and P2 adapters with barcodes and primers with indexes were designed according to Poland et al. (2012a), and pooled samples were size-selected for fragment sizes of 350–390 bp using a dye-free Blue Pippin 1.5% gel cassette (BDF1510) (Sage Science, Beverly, MA, USA). The sublibraries were pooled equimolarly and sequenced on the NovaSeq 6000 platform (Illumina) using the S1 reagent kit v.1.5 in 2 × 150 bp configuration at the Institute of Experimental Botany, Olomouc, Czechia.

The row reads of Mv9 (No. of reads 3,634,516; Sum. of length: 564.3 Mb; coverage of wheat genome: 0.039×), KAR (4,668,266; 732.3 Mb; 0.05×) and GLA8 (8,756,944; 1380.5 Mb; 0.095×) applied for the read coverage analysis using the low-pass genome-skimming approach described by Adhikari et al. (2022). Briefly, the chromosome-scale reference sequence of *Triticum aestivum* (IWGSC RefSeq v2.1 Chinese Spring) (Zhu et al. 2021) and *Thinopyrum intermedium* (A. glael's parental species) (*Thinopyrum intermedium* v3.1 DOE-JGI, http://phytozome.jgi.doe.gov/) were merged into a single file containing all 21 wheat chromosomes (A, B, D genomes) with all 21 *Thinopyrum* chromosomes (S, J, V genomes) enabling simultaneous detection of wheat and alien chromatin. Unlike SNP-based GBS analyses, no variant calling was performed. Instead, adapter and quality trimmed reads were aligned to the concatenated reference genomes with BWA-MEM (v0.7.17) under default parameters, followed by the removal of multi-mapped reads to exclude homeologous mis-assignments within and between the speces (i.e. wheat and *Thinopyrum*), depth normalization, and segmental coverage calculation for each chromosomes. Potential homoeologous mis-assignments were further minimized by evaluating coverage continuity across concatenated chromosomes. Coverage gaps on wheat chromosomes were interpreted as the absence of the chromosomal region in heterozygous (with values ~ 0.5) or homozygous (with values near 0) conditions, while coverage values greater than 0.2, approaching ~ 1.5 across alien chromosomes confirmed the presence of *Thinopyrum* chromatin.

### Field phenotyping investigations

The morphological traits of GLA8 and wheat parents Mv9 and KAR were investigated at one location, in the low-input, pesticide-free Tükrös nursery (Martonvásár, Hungary; geographic coordinates: 47°18′40″N 18°46′56″E) across three growth seasons (2021–2022, 2022–2023, 2023–2024). Herbicides, fungicides, and insecticides were not used in the low-input nursery. For each genotype, 40 grains were sown in 4 × 1 m rows, with 10 grains per row and 0.15 m spacing. Ten biological replicates from each genotype were randomly selected and phenotypically examined. To minimize the confounding effects of soil and microclimatic factors, genotypes were sown nearby. Plant height (cm), culm length (cm) and tillering (number of tillers per plant) were determined in the field before harvest. Following harvesting, the length of the main spike (cm), number of spikelets per main spike, number of grains per main spike, number of grains per spikelet (fertility), and number of grains per plant were all determined. The thousand-grain weight (g) was also measured. Additionally, the flowering date was recorded in all years of the experiments according to Tottman (1987).

### Investigation of plant development stages using Zadoks growth scale in greenhouse experiments

Ten biological replicates from the GLA8 line, as well as wheat parental genotypes Mv9 and KAR, were tested in greenhouse trials in 2024. Germinating seeds from the studied genotypes were planted in Jiffy pots with a diameter of 3 cm. Seedlings were vernalized at 4˚C for 6 weeks, then planted in 2 L pots with a 2:1:1 mix of garden soil, humus, and sand. The plants were grown till tillering at an initial temperature of 15˚C during the day and 10˚C at night, with a photoperiod of 12 h light and 12 h darkness. Temperature was raised by 2˚C during tillering (day length 14 h), stem elongation (16 h illumination), flowering, and 2 weeks after fertilization. Plant height and culm length (cm) measurements and Zadoks developmental stage determinations (Zadoks et al. 1974) for 10 plants from each genotype began two weeks after planting and were repeated every two weeks until complete ripening. At harvest, the ten biological replicates of each genotype were examined for the same morphological criteria as in field tests.

### Effect of gibberellic acid treatment on stem elongation at the seedling stage

Because the GLA8 genotype demonstrated decreased plant height in both field and greenhouse tests the sensitivity of this parameter to gibberellic acid (GA_3_) treatment was examined. The seeds of the three genotypes under investigation (KAR, Mv9, and GLA8) were germinated for 7 days at 25 °C between rolled filter sheets. The papers were soaked in either one-fourth- strength Hoagland solution (control) or Hoagland solution with 30 µM GA₃ for treatment. Each roll, holding 25 seeds, was placed upright in containers and sealed in a plastic bag. The used solutions were added to the bottom of the container to keep the rolls from drying out. Fifty seeds from each genotype and treatment (control and GA_3_) were tested. Throughout the entire process, the seeds were kept completely dark. After 7 days, the young plants' shoot and coleoptile length (cm) were measured.

The presence of dwarfing alleles of *Rht2* and *Rht8* (reduced height) genes in GLA8 genotype and wheat parents (KAR and Mv9) was validated using the PACE (PCR Allelic Competitive Extension) genotyping master mix (3CR bioscience, UK, Essex) as described by Rasheed et al. (2016) and Xu et al. (2024), respectively. Fluorescence-based detection of the reactions was carried out using the QuantStudio 5 system (Applied Biosystems, USA, California), and data analysis was conducted with the QuantStudio™ Design and Analysis Software version 1.5.0 (Applied Biosystems, USA, California).

### Shoot phenotyping of drought tolerance

The drought tolerance at the flowering stage of GLA8 and the parental wheat genotypes (Mv9 and KAR) was screened using a PlantScreen™ Modular high-throughput phenotyping system installed in Martonvásár by Photon System Instruments Ltd. PSI, Brno, Czech Republic. The different kinds of imaging sensors are physically separated from the growth area and each other. Before measurements, the plants were adapted to the light conditions used in the growth chamber. In case of fluorescence measurements, the effective quantum yield (EQY) parameter was determined with the use of 350 µmol m^−2^ s^−1^ actinic light intensity and 6700 µmol m^−2^ s^−1^ saturated pluse (1 s) for determination of Fm’. The EQY parameter was calculated according to the (Fm’-Fss)/Fm’ equation, where Fm′ means the maximum fluorescence yield in light-adapted state, and Fss means the steady-state fluorescence yield in light-adapted state The measurements, including the hyperspectral measurements, were performed during the morning period (between 08-10 h a.m.). Germinated seedlings were planted in Jiffy pots and vernalized at 4 °C for 6 weeks with low light intensity (20 µmol m^−2^ s^−1^). After vernalization, the plants were cultivated in 2L pots (1 plant/pot) loaded with a 3:2:1 mixture of garden soil, humus, and sand. The pots were arranged in 2 × 2 trays in a complete randomized blocks design, in a climate-controlled growing chamber (Walk-In FytoScope FS-WI, PSI, Brno, Czech Republic). Eight replications were used for each genotype. Until tillering, the plants were grown under a day/night temperature of 15/10 °C, photoperiod of 12/12 h, and light intensity of 250 µmol m^−2^ s^−1^. During the booting period, the day/night temperature and photoperiod were increased to 17/12 °C and 14/10 h, respectively, with 350 µmol m^−2^ s^−1^ of light intensity. During the flowering stage, the temperature was maintained at 19/16 °C with a photoperiod of 16/8 h and a light intensity of 350 µmol m^−2^ s^−1^. During the grain-filling period, the temperature was raised by 2 °C per week until harvest.

Drought stress was initiated at Zadoks developmental stage 41 (flag-leaf sheath extension), just before flowering began. The control plants were irrigated daily as before to maintain the optimal soil water content. At the control plants, the soil moisture content (SMWC) was kept at 33.4 ± 2.5 determined by a HH2 moisture meter (Delta T device AM-200 sensor, Delta T Device, Ltd., Cambridge, UK). Drought stress was induced by withholding water for 10 days, where the average daily SMWC was maintained at 10 ± 1,7 during the measurement period. Both control and drought-treated plants were automatically phenotyped using fluorescence, red–green–blue (RGB), infra-red and hyperspectral Visible-Near Infrared (VNIR) sensors of the PlantScreen™ Modular platform (PSI, Brno) on the 9th day of drought treatment. In the present paper, the effective quantum yield (EQY) parameter determined by chlorophyll fluorescence measurements at steady-state level of photosynthesis and the leaf (canopy) temperature (˚C) were determined according to Awlia et al. (2016). In addition, the NDVI (Normalized Difference Vegetation Index), reflecting plant greenness and density, and the mND705 (modified Normalized Difference Index), assessing leaf chlorophyll content, were determined based on reflectance spectra using the equations (R800 − R670)/(R800 + R670) and (R750 − R705)/(R750 + R705), respectively. Here, R denotes reflectance at a given wavelength (Rubo and Zinkernagel 2022; and Sims and Gamon 2002). From the RGB images, the relative percentage of different color categories was also determined as color segmentation data. The data and images were analyzed by PlantScreen™ software tool package (Data Analyser) according to the software instructions (PSI, Brno, Czech Republic). After high-throughput measurements, the flag-leaf segments were collected to determine relative water content (RWC) according to Darkó et al. (2020).

Following flowering, the plants’ initial water status was restored, and they continued to grow until ripening. After harvest, the culm length (cm), shoot weight (g), number of spikes per plant, number of spikelets per main spike, number of sterile spikelets per main spike, number of seeds per main spike, and yield per plant (number of seeds) were assigned. Grain length (mm), grain width (mm), and thousand-grain weight (TGW) were measured using a MARVIN 5.0 Seed Analyzer (MARViTECH, Wittenburg, Germany).

### Analysis of the expression patterns of drought-related genes

The expression of drought stress-related genes, *DEHYDRIN 3 (DHN3)*, *HEAT SHOCK PROTEIN 70 kDa (HSP70)*, *PLASMA MEMBRANE INTRINSIC PROTEIN 2 (PIP2)*, *TONOPLAST INTRINSIC PROTEIN 3 (TIP3)* and *4-COUMARATE-COA LIGASE (4CL)* were measured by Quantitative Reverse Transcription PCR (RT-qPCR). For RT-qPCR, three biological replicates were used for each genotype under each treatment, with three technical replicates per biological sample, resulting in a total of nine measurements per genotype per treatment. The RNA isolation from control and drought-treated flag-leaf samples of GLA8, KAR and Mv9, was carried out using the Direct-zol RNA MiniPrep Kit (Zymo Research, CA, USA). cDNA synthesis was done by an Invitrogen reverse transcription kit, using its recommended protocol (Invitrogen, Carlsbad, CA, USA). qPCR was done via qPCRBIO SyGreen Blue Mix (PCR Biosystem, London, UK), following the manufacturer’s protocol, on a CFX96 Touch Real-Time PCR Detection System (Bio-Rad Laboratories Inc, CA, USA). The used primers are set out in Supplementary Table 4. *GLYCERALDEHYDE-3-PHOSPHATE DEHYDROGENASE (GAPDH)* and *EF1 (ELONGATION FACTOR 1)* were picked as reference/constitutive genes, as they were deemed the most stable ones during these experimental conditions (the third reference gene, *ACTIN (ACT)* showed a minor response to the stress; therefore it was excluded). For primer efficiency calculations, LinRegPCR was used (Ramakers et al. 2003). The calculation of relative expressions was done by the Pfaffl method (Pfaffl 2001). For the creation of heatmap and clustermap Python (v3.12) software and the pandas (v2.2.3), numpy (2.1.1), seaborn (0.13.2), matplotlib (3.9.2) and scipy (1.14.1) libraries were used.

### Determination of root development under drought stress

The root parameters of the GLA8 line and its wheat parents (Mv9 and KAR) were investigated in a sand tube experimental setup under climate-controlled greenhouse conditions. After 6 weeks of vernalization, the seedlings were planted in sand-filled PVC tubes (80% sand particle size between 0.18 and 0.35 mm) with a height of 75 cm and 11 cm in diameter. Each tube held one well-developed seedling. The plants were cultivated in a greenhouse under controlled climatic circumstances, following Tischner et al.'s (1997) spring–summer climate program. The plants were watered three times a week with a half-strength Hoagland solution. The control plants received water throughout the growth season. In the drought stress treatment, water shortage was simulated at Zadoks developmental stage 31 (first detectable node) (Zadoks et al. 1974). Following 21 days of full water removal, the drought treatment plants were re-watered with Hoagland solution. At the hard dough ripening stage (Zadoks 87), the plants were removed from the plastic tubes and the root system was thoroughly cleansed with tap water. Root sampling was performed across the full tube depth (0–75 cm). No additional staining was applied before imaging. The total root length, diameter, root area and volume of the complete root system were measured using the WinRHIZO Pro root analysis equipment (Regent Instruments Ltd., Canada). WinRHIZO image analysis was carried out on images acquired with an Epson P. V85 Pro scanner at 800 dpi resolution. Root detection was based on gray level with automatic threshold selection (threshold ≈128), and roots were defined as darker than the background. A minimum region size of 44 pixels was applied. Debris was excluded using a length/width ratio filter (objects with a ratio smaller than 4 were discarded), and rough edge removal was set to medium. These settings follow the manufacturer’s recommendations, ensuring reproducible measurements of root traits. During scanning, roots were spread in distilled water, which facilitated separation and provided better image contrast. In addition, the surface-to-volume ratio was calculated as the ratio between the total surface area and the total volume of roots, reflecting whether roots tend to be finer (higher ratio) or thicker (lower ratio) under stress conditions. Following root parameter detection, the roots were dried in an oven at 70 °C for 48 h, and the root dry weight was determined using a balance (ICS689g-A15, Mettler Toledo Ltd., Budapest, Hungary). Each treatment was represented by four biological replicates in the sand tube experiment.

### Statistical analysis

At least 8–10 biological replicates per genotype and treatment were used in each experiment performed in the field, glasshouse, and indoor phenotyping system. Tukey’s post hoc test was used to identify statistically significant differences between means of GLA8 and the wheat parents (Mv9 and KAR) within one experiment, where different letters indicated significant differences at the *p* ≤ 0.05 level. The effect of genotypes (*A*), environments (*B*), and their combinations (*A* × *B*) were calculated by two-way ANOVA, where different years were considered as different environments, using IBM SPSS Statistics 20.0 software (SPSS Inc., Chicago, IL, USA).

## Results

### Chromosomal composition of the GLA8 line

**Fig. 2:**
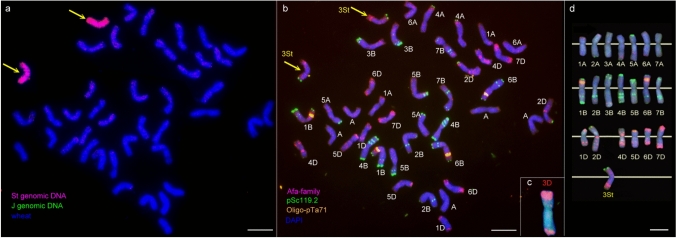
colour figure online Genomic *in situ* hybridization (GISH) (**a**) and fluorescence *in situ* hybridization (FISH) (**b**) on a mitotic metaphasic cell from the stable GLA8 line: **a** mcGISH on the metaphase cell: *Pseudoroegneria spicata* (2*n* = 2*x* = 14, St) (red) and *Thinopyrum bessarabicum* (2*n* = 2*x* = 14, J^b^) (green) were used as labeled DNA probes. The St chromatine is visualized in red (yellow arrows). No hybridization signals could be detected with the J^b^ probe while the two probes (St and J^b^) were used simultaneously. DAPI-stained wheat chromosomes are blue. Scale bar = 10 μm. **b** FISH patterns using DNA repetitive probes: Afa-family (red), pSc119.2 (green), oligo-pTa71(yellow) of the same cell. Forty wheat chromosomes and the pair of St chromosomes have been identified. Scale bar = 10 μm. **c** FISH pattern of missing chromosome pair 3D, which was replaced by a pair of 3St. **d** FISH karyogram displaying the hybridization sites of oligo-pTa71, pSc119.2, and Afa-family probes on all wheat chromosomes (except 3D) and the *Thinopyrum* chromosome (white lines indicate centromere positions). Scale bar = 10 μm

The chromosomal content of the GLA8 substitution line was determined using the mcGISH technique (Fig. 2). Chromosomes of A. glael were identified using fluorescently labeled genomic DNA from *Pseudoroegneria spicata* (2*n* = 2*x* = 14, StSt) and *Thinopyrum bessarabicum* (2*n* = 2*x* = 14, J^b^J^b^). In the BC_2_F_7_ generation, a genotype with 42 chromosomes, 40 wheat and 2 originating from the St genome was identified, as evidenced by consistent red signals visible along 2 chromosomes and the absence of red or green fluorescent signals on 40 chromosomes (Fig. 2a). J genome's chromosomes could not be identified while the two probes (St and J^b^) were used simultaneously. Following GISH, the fluorescence signals were removed and the slides were subjected to FISH analysis utilizing repetitive DNA probes Afa-family (red), pSc119.2 (green), and oligo-pTa71 (yellow). Forty wheat chromosomes were detected as well as the absence of the 3D chromosome (Fig. 2b–d), while the St genomic chromosomes showed a particular FISH pattern which consisted of a terminal pSc119.2 (green) signal on the short arm and a subterminal Afa-family (red) signal on the long arm (Fig. 2b yellow arrows). FISH with the (GAA)_7_ microsatellite probe produced no fluorescence signal (image not shown). *In situ* hybridization results confirmed the presence of a pair of St genome chromosomes. This line's cytogenetic stability is outstanding; monosomic substitutions or wheat telosomics were exceedingly rarely detected, while almost all examined plants carried the St pair of chromosomes in disomic form.

### GBS read coverage analysis

GBS read coverage analysis was conducted by aligning filtered read data from the GLA8 genotypes to the combined *T. aestivum*-*Th. intermedium* reference genome to determine the chromosome composition of the substitution line and to evaluate whether the introgressed *Thinopyrum* chromosome is homoeologous to the missing wheat chromosome.

**Fig. 3:**
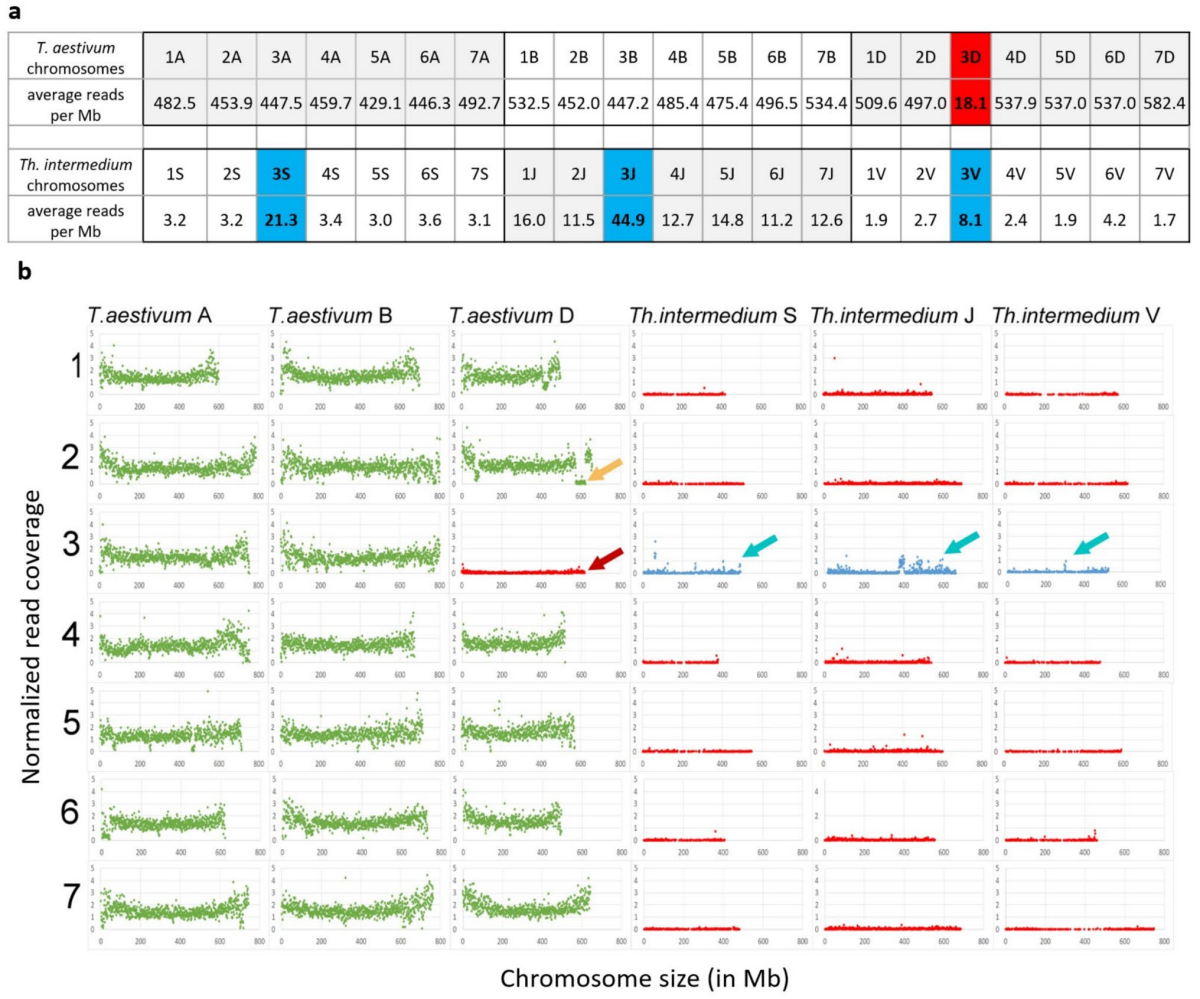
colour figure online **a** Mean read density (reads per megabase bin) in the GLA8 wheat-wheatgrass substitution line, mapped across the chromosomes of the *T. aestivum* and *Th. intermedium* reference genomes. **b** Normalized GBS read coverage of the GLA8 substitution line across chromosomes 1–7 of the wheat A, B, and D subgenomes, and the S (St), J (J^r^), and V (J^vs^) genomes of *Thinopyrum intermedium*. The *x* axis indicates genomic position (Mb), and the y-axis shows normalized read coverage. Red arrow: absence of chromosome 3D; yellow arrow: previously reported *Aegilops ventricosa* introgression on chromosome 2D; cyan arrows: *Thinopyrum*-derived chromosomal segments

Read density across the A, B, and D subgenomes of wheat ranged from 429.1 to 582.4 reads per megabase (Fig. 3a). In stark contrast, chromosome 3D exhibited a markedly reduced read density of only 18.1 reads per megabase-corresponding to a 95.8–96.9% decrease relative to the other chromosomes. This substantial depletion strongly supports the absence of chromosome 3D in the GLA8 genotype, in line with the molecular cytogenetic results. The unique read density of GLA8 on group 1, 2, 4, 5, 6 and 7 on the S, J, and V in silico chromosomes averaged 3.3, 13.2, and 2.5 reads per Mb, respectively. In contrast, group 3 chromosomes displayed significantly higher densities, with 21.3, 44.9, and 8.1 unique reads per 1 Mb on the 3S, 3 J, and 3 V chromosomes, respectively-indicating an approximately fourfold increase on chromosomes 3 J and 3 V and a sevenfold increase on chromosome 3S compared to other homoeologous group chromosomes (Fig. 3a). A deviation of this magnitude strongly suggests that the introgressed *Thinopyrum* chromosome belongs to homoeologous group 3.

Following normalization and graphical visualization of read distribution, the majority of chromosomes displayed normalized read values between 1 and 3 per data point (Fig. 3b). In contrast, chromosome 3D was represented by a continuous, line-like distribution of data points with normalized values ranging from 0 to 0.1, providing additional evidence for its absence in the GLA8 line (marked by the red arrow in Fig. 3b). Low normalized read values were found on the long arm of chromosome 2D, ranging from 574 to 621 Mb (marked by the yellow arrow in Fig. 3b), indicating the presence of a previously described introgression from *Aegilops ventricosa* (Laugerotte et al. 2022) and present in several wheat cultivars including Mv9 and KAR wheat genotypes.

In addition to the identification of wheat chromosomes present in the substitution line, GBS read coverage data provided information about the presence of *Thinopyrum* chromosomes. The normalized read coverage values across the reference chromosomes of J (J^r^), S (St), and V (J^vs^) genomes were consistently below 0.1, indicating the absence of *Thinopyrum* homoeologous group 1, 2, 4, 5, 6, and 7 chromosomes. However, a distinct profile was observed along the *Thinopyrum* group 3 chromosomes (marked by cyan arrows in Fig. 3b). The results obtained with the GISH technique confirm the presence of a St chromosome in the investigated substitution line; however, this was not clearly corroborated by the GBS read coverage analysis. Since Agropyron glael is derived not only from *Th. intermedium* but also from *Th. ponticum*, we sought to incorporate a genome in our analysis that might offer a closer comparison to *Th. ponticum*. Using reference sequences of *Th. elongatum*, another close relative of Agropyron glael, we were able to further support the presence of a *Thinopyrum* chromosome belonging to the homoeologous group 3. Thus, an *in silico* hybrid of wheat and *Th. elongatum* was used to expand the analysis. GLA8 line shows an average of only 10.6 unique reads per 1 Mb on *in silico* 1, 2, 4, 5, 6, and 7E chromosomes, whereas a threefold increase (27.5) was detected on chromosome 3E (data not shown). This supports the hypothesis that the wheat chromosome 3D was replaced by a group 3 *Thinopyrum* chromosome.

### Field and greenhouse phenotyping investigations

The morphological traits of GLA8 and the crossing partner wheat genotypes (Mv9, KAR) were compared in three-year field trials. The weather conditions during the experiments were largely similar to the Central European average. Between sowing and harvest, precipitation was 293 mm (2021–2022), 430 mm (2022–2023), and 460 mm (2023–2024), with 78 mm, 105 mm, and 120 mm dropping during the grain-filling period. Heat days (temperatures above 30 °C) were 9 in 2022, 5 in 2023, and 7 in 2024, till harvesting. The effects of genotype, environment (years), and their interaction were calculated by two-way ANOVA on the measured traits and summarized in Supplementary Tables 1–3. Genotype had a significant effect on plant height, tiller number, spike length, seed number on the main spike, fertility, and culm length. Environment significantly influenced plant height, tiller number, spike length, spikelet number on the main spike, yield, and culm length. A significant genotype × environment interaction was found only for tiller number. Trends toward significance were observed for spikelet number on the main spike and yield.

**Fig. 4:**
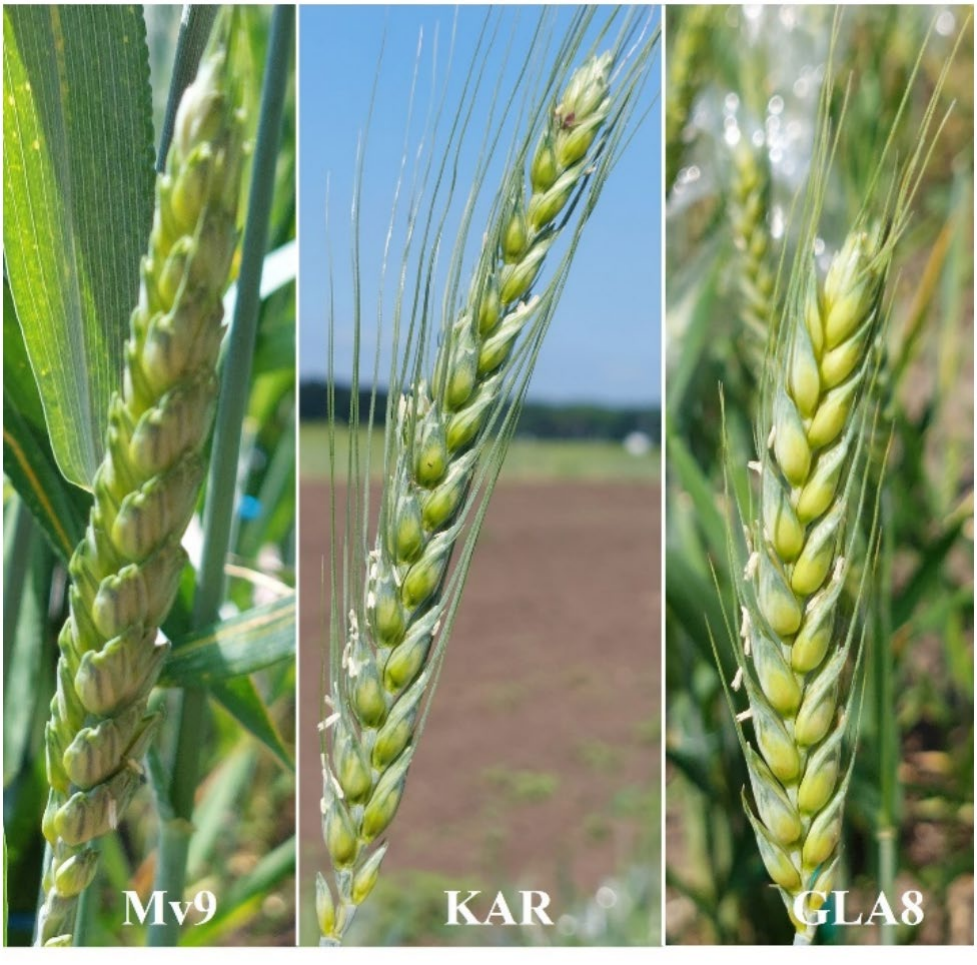
Spike morphology of the wheat controls (Mv9 and KAR wheat genotypes) and that of the GLA8 line (Tükrös nursery, Martonvásár, Hungary, May 2024)

Table 1 presents the findings from the field investigations (data on the phenotypic characteristics of plants cultivated in the greenhouse are also included). Tukey’s post hoc comparisons within each experiment (Table 1) showed that GLA8 line grew much shorter than the wheat control genotypes in all growing seasons and greenhouse trials, with no plant height exceeding 65 cm. Until now, no wheat/A. glael introgression lines exhibited this semidwarf habit. In GLA8, the reduction in total plant height relative to the parental lines was accompanied by a decrease in culm length across all trial sites, and this decrease was statistically significant compared to both parents in each of the three years at the Tükrös nursery. In all experiments, the number of tillers per plant for GLA8 was slightly higher than that of wheat parents, with the difference being significant during the 2023–2024 growth season and greenhouse trial. The GLA8 line exhibited the shortest spikes, with significantly lower values in every year and greenhouse (Fig. 4), although the number of spikelets per spike and number of seeds per main spike were comparable to wheat genotypes due to the spikelets' denser position on the rachis. Only in the greenhouse trial were these values significantly lower for the GLA8 line than those of the wheat parents. The mean fertility (number of grains per spikelet) of the GLA8 was greater than 1.80 in all experiments, while the number of grains per plant was similar to that of wheat genotypes. In contrast, in the greenhouse, the number of grains per plant was significantly higher than that of the wheat parents due to the substitution line's significantly better tillering. Except for the 2023 and 2024 field testing, the GLA8 line produced smaller seeds that had significantly lower TGW than both wheat parents.

**Table 1.** Morphological traits of the GLA8 line compared to the parental wheat genotypes (Mv9 and KAR) in three vegetative seasons (2021–2022, 2022–2023, 2023–2024 and the greenhouse trial, respectively)

Location	Genotype	Plant height (cm)	Culm length (cm)	Number of spikes per plant	Length of main spike (cm)	No. of spikelets per main spike	No. of grains per main spike	Fertility (grains per spikelet)	Number of grains per plant	Thousand-grain weight (g)
Tükrös nursery 2022	*Mv9*	87.8 ± 5.8^a^	78.8 ± 5.3^a^	8.2 ± 2.2^a^	9.0 ± 1.1^b^	18.3 ± 1.7^a^	47.6 ± 6.9^a^	2.6 ± 0.2^a^	366 ± 82^a^	39.61 ± 2.6^a^
	*KAR*	81.3 ± 3.6^b^	71.2 ± 3.6^b^	8.7 ± 2.0^a^	10.2 ± 0.4^a^	19.2 ± 0.8^a^	39.0 ± 2.9^b^	2.0 ± 0.2^b^	291 ± 72^a^	42.83 ± 3.7^a^
	*GLA8*	**62.6 ± 4.1**^**c**^	**55.2 ± 4.2**^**c**^	9.2 ± 1.9^a^	**7.4 ± 0.7**^**c**^	19.6 ± 1.3^a^	42.4 ± 10.9^ab^	2.2 ± 0.5^b^	339 ± 101^a^	**33.7 ± 0.9**^**b**^
Tükrös nursery 2023	*Mv9*	84.4 ± 9.6^a^	73.2 ± 9.5^a^	6.5 ± 3.0^a^	11.2 ± 1.4^b^	22.0 ± 1.9^a^	55.6 ± 13.9^a^	2.5 ± 0.5^a^	279 ± 145^a^	40.8 ± 3.0^a^
	*KAR*	79.2 ± 4.0^a^	67.3 ± 4.3^a^	8.2 ± 2.8^a^	11.9 ± 1.7^a^	20.9 ± 1.4^a^	42.6 ± 16.0^a^	2.0 ± 0.7^a^	284 ± 124^a^	36.5 ± 4.0^b^
	*GLA8*	**60.9 ± 3.0**^**b**^	**52.6 ± 3.0**^**b**^	8.5 ± 2.1^a^	8.4 ± 0.9^b^	20.5 ± 0.5^a^	42.8 ± 9.6^a^	2.1 ± 0.4^a^	205 ± 56^a^	36.5 ± 1.2^b^
Tükrös nursery 2024	*Mv9*	90.0 ± 2.6^a^	80.0 ± 3.0^a^	5.0 ± 1.5^b^	10.0 ± 1.0^a^	21.7 ± 1.5^a^	53.1 ± 13.0^a^	2.4 ± 0.5^a^	214 ± 81^ab^	48.9 ± 1.3^a^
	*KAR*	88.8 ± 5.6^a^	78.5 ± 5.0^a^	4.2 ± 1.3^b^	10.2 ± 1.1^a^	20.8 ± 2.4^a^	46.6 ± 10.8^ab^	2.3 ± 0.5^ab^	163 ± 65^b^	44.9 ± 2.4^b^
	*GLA8*	**64.1 ± 4.3**^**b**^	**56.4 ± 3.9**^**b**^	**8.7 ± 1.8**^**a**^	**7.7 ± 0.8**^**b**^	21.6 ± 1.7^a^	40.1 ± 9.4^b^	1.9 ± 0.4^b^	249 ± 59^a^	33.09 ± 2.5^b^
Green house 2024	*Mv9*	61.9 ± 8.0^a^	53.7 ± 8.7^a^	1.1 ± 0.3^b^	8.2 ± 0.9^b^	19.6 ± 2.0^a^	38.7 ± 10.9^a^	2.0 ± 0.4^b^	38.7 ± 10.9^b^	35.91 ± 1.9^a^
	*KAR*	54.1 ± 5.6^b^	44.5 ± 5.9^b^	1.0 ± 0.0^b^	9.7 ± 0.4^a^	18.1 ± 0.6^a^	44.7 ± 2.4^a^	2.5 ± 0.2^a^	44.7 ± 2.4^b^	34.12 ± 1.4^a^
	*GLA8*	**44.0 ± 3.7**^**c**^	37.7 ± 3.3^b^	**3.4 ± 0.5**^**a**^	**6.3 ± 0.6**^**c**^	**16.3 ± 1.4**^**b**^	**28.8 ± 6.5**^**b**^	1.8 ± 0.4^b^	**68.6 ± 6.6**^**a**^	**26.61 ± 1.7**^**b**^

Data are means of ten measurements ± standard deviation. Tukey’s post hoc tests were performed within each experiment to determine significant differences between the genotypes. Different letters indicate significant differences at *p* ≤ .0.05 level. The values in bold show that GLA8 differed significantly from both wheat parents.

Regarding the flowering time, generally wheat/A. glael introgression lines exhibit a significantly delayed flowering compared to wheat progenitor genotypes (data not reported). The GLA8 line has an early heading, implying an earlier flowering time. During field experiments, the average flowering time (days after 1 January, average of three vegetative seasons) was 131 days for Mv9, 135 days for KAR, and 129 days for GLA8. The section "Comparison of plant height and Zadoks developmental phases of the GLA8 line to wheat parental genotypes" provides information on the flowering time of the genotypes recorded in greenhouse studies. In both the field trials and the greenhouse experiment, the substitution line had earlier flowering times than the controls. In repeated trials, the GLA8 line's morphological traits were comparable to those of the parent lines, albeit the substitution line exhibited a semidwarf stature, improved tillering, significantly shorter spikes, and lower TGW.

### Comparison of plant height and Zadoks developmental phases of the GLA8 line with wheat parental genotypes

**Fig. 5:**
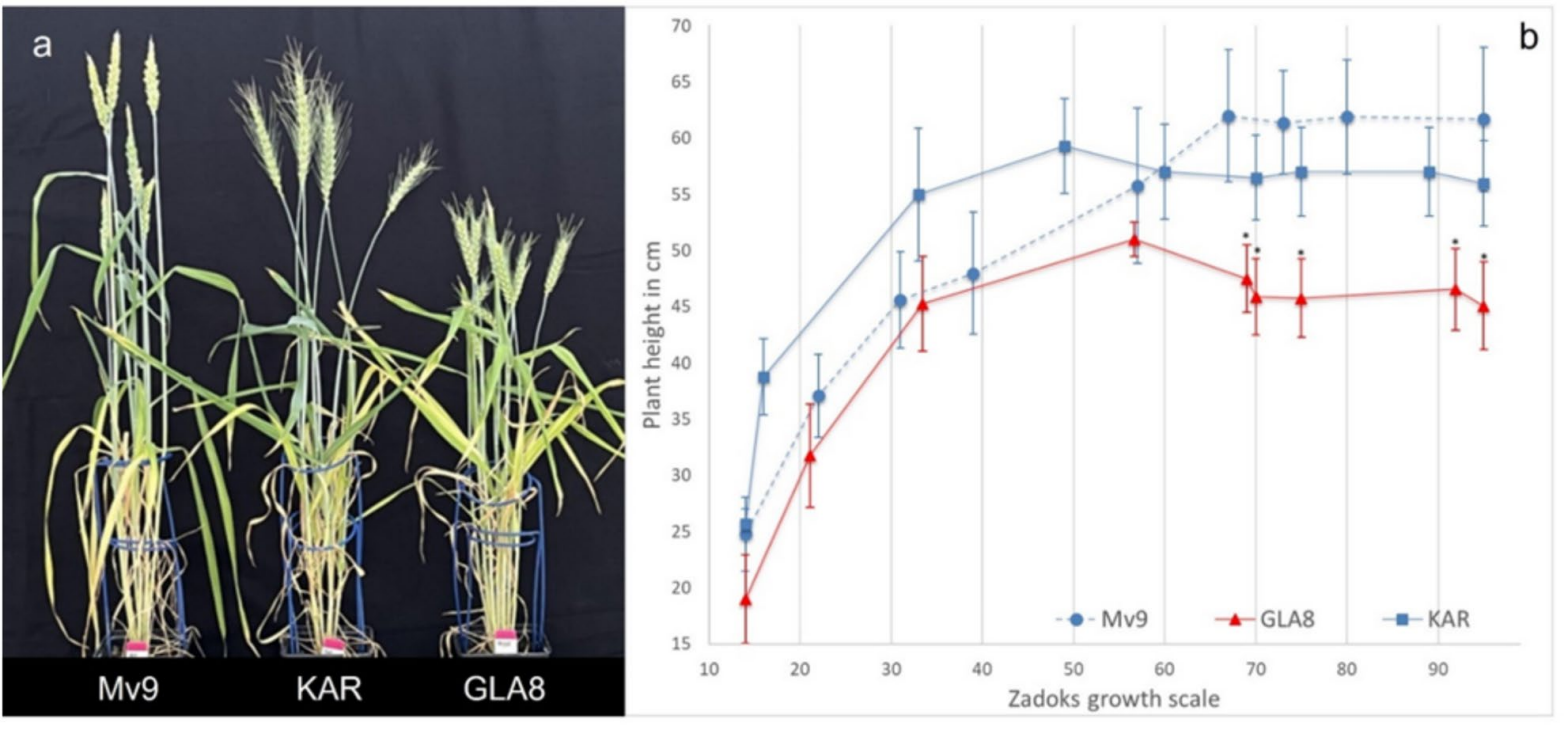
**a** Phenotypes of Mv9, KAR and GLA8 (from left to right) grown in the greenhouse (Martonvásár, Hungary, June 2024). **b** Developmental time-course of plant height at the investigated genotypes (Mv9, KAR, GLA8 line) grown under greenhouse conditions. Data points combine measurements from 10 individuals of each genotype. Vertical bars represent standard deviation. The plant height was significantly different for GLA8 line from the measurements marked with an asterisk

In a greenhouse experiment, the GLA8 line and control wheat genotypes (Mv9 and KAR) were compared in terms of plant height (Fig. 5a) and Zadoks development phases (Fig. 5b). Every two weeks, plant height measurements and developmental stage determinations were performed on (the same) ten biological replicates of each genotype. The mean values of the GLA8 plant height at all developmental stages were lower than those of the parental genotypes. Starting with the fifth measurement, the difference in plant height between the GLA8 line and the parents was statistically significant. GLA8 individuals reached the flowering developmental stage (Zadoks 60–65) two weeks earlier (flowering between the fourth and fifth measurements) than Mv9 and KAR. At the penultimate measurement, the GLA8 plants were ready to harvest (Z92 stage, seed hard) while the wheat cultivars were at the Z80 (early dough) stage (Mv9) and Z87 (hard dough) stage (KAR).

### Effect of GA treatment on stem elongation at the seedling stage

**Fig. 6:**
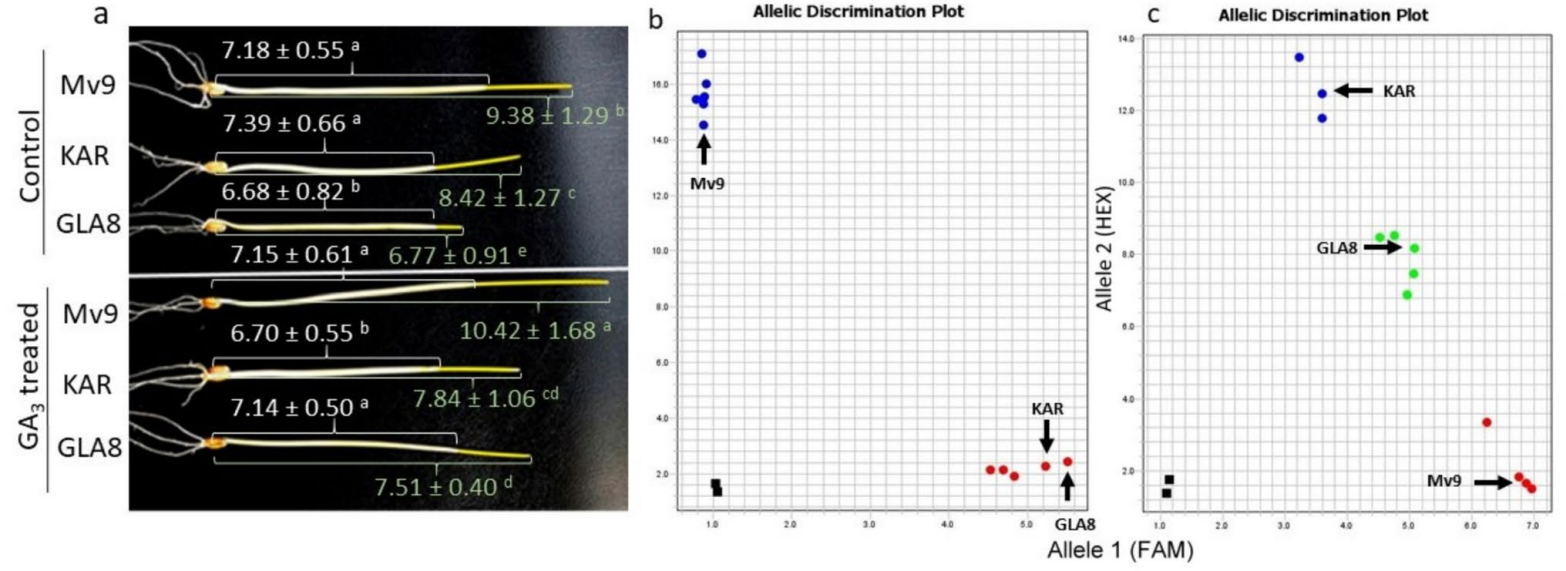
colour figure online **a** GA_3_-treated and control seedlings of the genotypes under analysis: GLA8 substitution line and wheat genotypes Mv9 and KAR. For every treatment and genotype, the mean values and standard deviation are indicated. Values with the same letter do not differ significantly, according to Tukey's post hoc test (*α* = 0.05). **b** and **c** Allelic discrimination plot for validating the presence of *Rht2* and *Rht8* dwarfing alleles in GLA8 substitution line and its parental wheat lines **b** Rht-D1_SNP primers demonstrated the presence of the *Rht2* dwarfing allele in the GLA8 genotype and KAR control wheat cultivar in homozygous form (red dots) while in Mv9 line the wild type allele (*rht2*) was present (blue dots) **c** Rht8-KASP-V3 marker validated the presence of *Rht8* dwarfing allele in Mv9 wheat line in homozygous form (red dots) and in GLA8 at least in heterozygous form (green dots) while KAR cultivar carries the wild type allele (*rht8*) (blue dots). The SDS software graphs the results of allelic discrimination runs on a scatterplot (the Allelic Discrimination Plot) that contrasts reporter dye fluorescence. After signal normalization and multicomponent analysis, the SDS software graphs the normalized data from each well as a single datapoint on the scatterplot

In the control treatment, the GLA8 had the shortest shoot and coleoptile, as expected given adult plant measurements. After treating the seeds with GA_3_ for a week, the shoot lengthiness increased significantly for the Mv9 and GLA8 genotypes, while for the KAR wheat cultivar, there was no significant difference. Only the GLA8 substitution line resulted in a significant increase in coleoptile length after GA_3_ treatment. Figure 6a shows the results of the GA_3_ treatment at the seedling stage. Because both wheat parents carry different dwarfing genes (KAR, the *Rht2* GA-insensitive allele, and Mv9, the *Rht8* GA-sensitive allele), it was thought that GLA8's semidwarf habit was caused by the additive effect of the two dwarfing genes. This is corroborated by GA_3_ treatment results at the seedling stage as well as PACE (PCR Allelic Competitive Extension) marker analysis of the three genotypes. Figure 6b, c shows the allelic discrimination plot for validating the presence of *Rht2* and *Rht8* dwarfing alleles in GLA8 substitution line and its parental wheat lines.

### Shoot phenotypic traits under drought stress

**Fig. 7:**
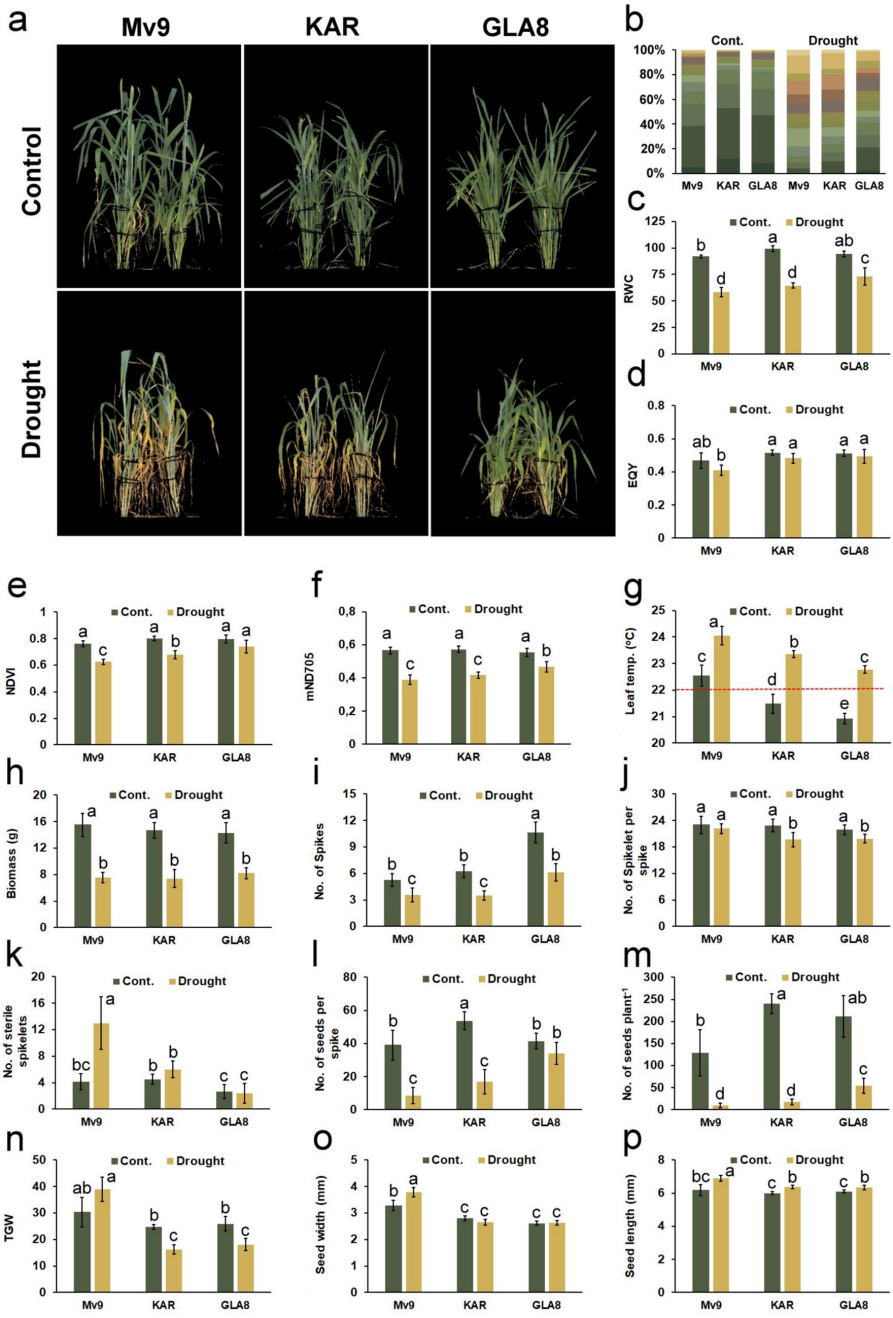
Results of the drought tolerance tests for Mv9 and KAR wheat parents, as well as the GLA8 substitution line: **a** Control and drought-stressed plants were automatically phenotyped for RGB traits using the PlantScreen™ platform, both before and after drought treatment. **b** Leaf color segmentation for each genotype under control and drought conditions was performed using RGB pixel image analysis. The relative percentages of different colors are presented. Mean values for relative water content RWC % (**c**), effective quantum yield EQY (**d**), Normalized Difference vegetation Index NDVI (**e**), modified Normalized Difference Index 705 mND705 (**f**), leaf (canopy) temperature (˚C) (**g**), shoot weight in grams (**h**), number of spikes per plant (**i**), number of spikelets per main spike (**j**), number of sterile spikelets per main spike (**k**), number of seeds per main spike (**l**), number of seeds per plant (**m**), thousand-grain weight TGW in grams (**n**), grain width in mm (**o**), and grain length in mm (**p**) for each genotype. Standard errors of the mean are shown as error bars. Two-way ANOVA with Tukey’s post hoc tests were performed for each genotype across all yield parameters under both control and drought stress treatments (*α* = 0.05, *n* = 8). Different letters above the error bars indicate statistically significant differences between treatments (Cont. = Control)

During the 10-day drought stress period, which began at Zadoks 41 stage, both the control and drought-treated plants were headed. Figure 7a–p shows the results of morphological and physiological traits measured on control and treated plants.

Culm length showed a clear reduction under drought conditions compared with the control in all three tested genotypes, with this reduction being statistically significant for each genotype. Under control conditions, the genotypes differed significantly from each other, with Mv9 exhibiting the highest mean culm length (61.9 ± 3.0 cm), KAR showing intermediate values (57.4 ± 1.8 cm), and GLA8 having the lowest (46.7 ± 1.9 cm). Under drought, however, the culm length of GLA8 did not differ significantly from KAR (37.8 ± 4.8 cm vs. 39.6 ± 2.1 cm), indicating that the reduction in culm length due to drought was less pronounced in GLA8 compared with the other genotypes. Mv9 still showed the highest height under drought (44.3 ± 1.6 cm) (Supplementary Fig. 1a).Leaf senescence was less pronounced at GLA8 after drought stress treatment than at the parental wheat genotypes (Fig. 7a, b), with the “stay-green” trait being an excellent indicator of drought tolerance. This feature was investigated by the plant phenotyping system, which converted the plant greenness trait into a color segmentation image using RGB sensors. After withholding water, the wheat parents' shoots had a larger percentage of yellow portions than those of the GLA8 line (Fig. 7b). The adverse effect of drought stress was also manifested in the decrease of NDVI and mND705 reflectance indices (Fig. 7e, f) and in the increase of average canopy temperature (Fig. 7g). However, the drought-induced changes were less pronounced in the GLA8 genotype as compared to wheat Mv9 and KAR genotypes. Furthermore, drought treatment reduced the RWC of leaves significantly less in the GLA8 line (23%) than in wheat parental genotypes (36% in Mv9 and 35% in KAR, respectively) (Fig. 7c). It should be noted however, that the changes in EQY chlorophyll *a* fluorescence parameter were less pronounced (Fig. 7d), than those mentioned above, implying that this parameter is less sensitive for discriminating drought stress responses.

The effect of drought stress on the examined genotypes was monitored after plant harvest, when biomass (g) and yield-related characteristics were evaluated. In terms of aboveground biomass, all three genotypes showed a significant decline, with no significant differences between genotypes in the extent of the decrease after drought treatment (Fig. 7h). The similar decline in tiller number was detected for treated plants of all genotypes, however GLA8 had a significantly higher number of tillers than wheat parents in both control and drought treatment (Fig. 7i), probably because earlier development and tiller formation. The number of spikelets per main spike, sterile spikelets per main spike, and seeds per main spike were determined for each examined plant (Fig. 7j–l). At the treated plants, the number of spikelets per main spike lessened significantly for KAR and GLA8, while Mv9 remained similar to the control (Fig. 7j). The number of sterile spikelets per main spike increased significantly for Mv9 and slightly for KAR, while for GLA8 remained constant in stressed plants compared to well-watered ones (Fig. 7l). The previous three traits resulted in a significantly reduced yield per plant following stress for all genotypes, the GLA8 line having significantly higher number of grain per plant (Fig. 7m) and yield (weight) per plant than the parents (Supplementary Fig. 1b). Following drought stress, TGW increased for Mv9 and reduced for KAR and GLA8 (Fig. 7n), however when all prior measurements of plant yield were taken into account, the GLA8 line had the least yield decrease. Seed width (mm) and length (mm) were measured as well at the genotypes under consideration. Seed width rose considerably only for Mv9 after drought stress, although seed length increased significantly for all three genotypes. After the water supply was restored, the decrease in the number of grains caused by drought stress was compensated for by an increase in the length of the grains. Notwithstanding, the earlier flowering GLA8 showed less yield loss compared to the parents after applying severe drought stress at the flowering stage. According to the changes in morphological, physiological, and yield-related parameters, withholding watering for 10 days at the flowering stage caused more severe drought stress for wheat genotypes (Mv9 and KAR) than for the GLA8 substitution line, as shown by the results presented in Fig. 7.

### Analysis of the expression patterns of drought-related genes

To characterize the intensity of some cellular defending mechanisms under drought stress, the expression level of genes known to be involved in membrane and protein stabilization (*DHN3*, *HSP70*), flavonoid accumulation (*4CL*), and inter- and intracellular transport of water and small molecules (*PIP2*, *TIP3*) were investigated.

**Fig. 8:**
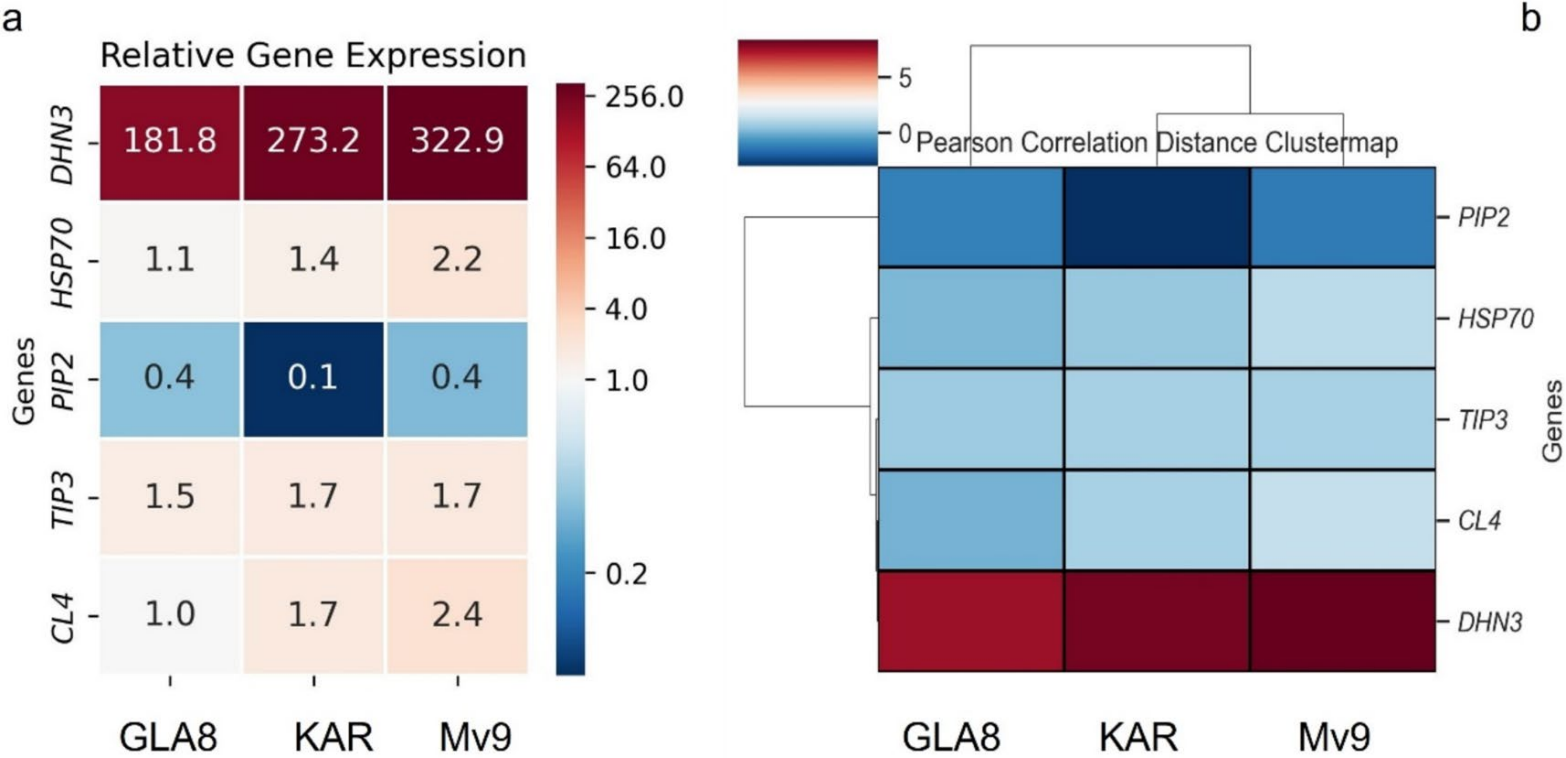
**a** Relative expression of the investigated genes: *DHN3, HSP70, PIP2, TIP3* and *4CL* in the GLA8 substitution line and the parental wheat genotypes KAR and Mv9. The cells are annotated with the actual relative expression values, obtained via the Pfaffl method. **b** Distance clustermap of the investigated gene expressions via Pearson correlation. Coloration indicates the log_2_ value of the expression via the Pfaffl method

*DHN3* showed an extremely high activation due to stress, where the relative expression increased by ~ 323 times in Mv9, ~ 273 times in KAR, and ~ 182 times in GLA8. On the other hand *HSP70* remained unaffected in KAR and GLA8, but increased by ~ 2 times in Mv9. *PIP2* exhibited downregulation in both Mv9 and GLA8 (~ 0.4), while the decrease was more extreme in KAR (~ 0.1). However, *TIP3* expression was relatively unaffected across the genotypes, where GLA8 exhibited the mildest change. *4CL* was also unaffected in GLA8, while in KAR it was only mildly affected. On the other hand, in Mv9, it showed a ~ 2.4 times upregulation compared to control (Fig. 8a).

The *DHN3* expression pattern shows that, in contrast to KAR and Mv9, GLA8 was the most resilient to the stress. In all genotypes, the expression of *DHN3* was high, but in GLA8, its expression increased the least. This is also supported by *HSP70* expression, where the same pattern was seen, albeit to a lower degree. To maintain appropriate cellular functions, the cells actively sought to preserve water, as seen by the significantly reduced *PIP2* and slightly increased *TIP3* expressions. The degree of these aquaporins' expression also indicates that GLA8 was the most resilient to these conditions, as evidenced by the fact that these genes’ expressions changed the least. This assertion is further supported by expression of *4CL*, which revealed increased levels in KAR and Mv9 but no change in GLA8 (Fig. 8a). Clustering via Pearson correlation also resulted in pairing the KAR and Mv9 first and linking GLA8 after, showing that at expressional levels GLA8 exhibited different characteristics (Fig. 8b).

### Changes in the root morphology under drought stress

**Fig. 9:**
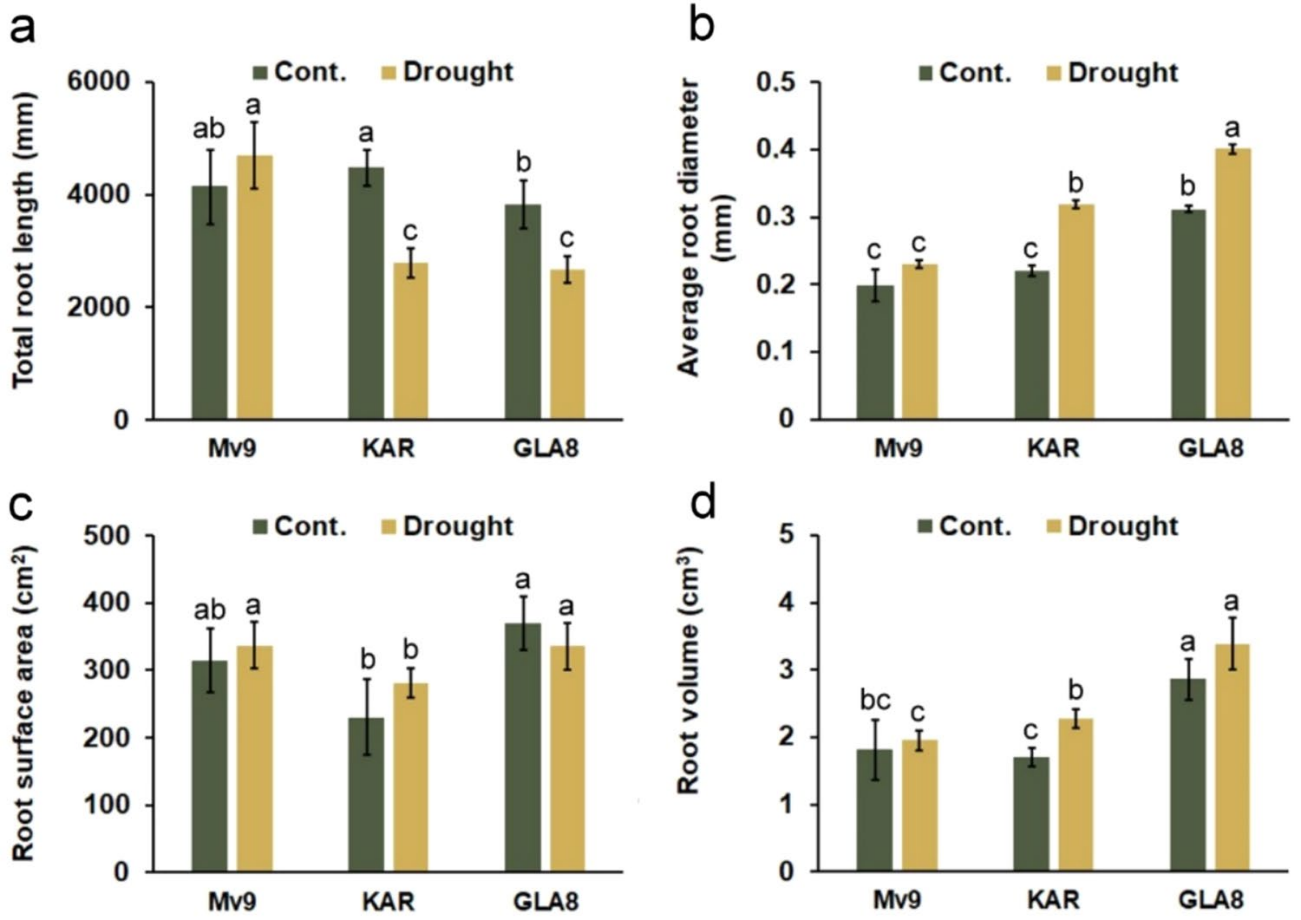
colour figure online Effects of the water shortage on **a** the total root length, **b** average root diameter, **c** root surface area and **d** total root volume of Mv9 wheat line, KAR facultative wheat cultivar and the GLA8 substitution line (control = left column, in green; drought stress = right column, in yellow). Different letters above the error bars indicate statistically significant differences between treatments (*α* = 0.05). Vertical bars represent standard deviation (Cont. = Control)

The analysis of variance shows that there were significant differences under well-watered conditions between GLA8 and both parental genotypes (Mv9 and KAR) regarding average root diameter and total root volume (Fig. 9). The water shortage resulted in a decrease in the total root length of KAR and GLA8 line (38.7 and 30.7%, respectively) compared with the well-watered control. However, for Mv9, the total root length slightly increased during drought stress. Under drought-stressed conditions, the root length of Mv9 was significantly higher than at the other two genotypes, while no significant differences could be detected between KAR and GLA8 lines. The average root diameter of Mv9 did not show a change as a result of the water withdrawal compared to the control; however, a significant increase in average root diameter (45.4 and 28.7%, respectively) was measured for KAR and GLA8. In a water-limited environment, GLA8 had a significantly larger root diameter than the other two genotypes, and even the root diameter of Mv9 was significantly lower than that of KAR. The effect of the genotype on the root surface area was not significant either under optimum irrigation or under simulated drought stress conditions (the same treatment), and no significant impact of the watering levels can be confirmed statistically between the two watering levels. The root volume of GLA8 was significantly higher under optimum watering than that of KAR and Mv9. Only for KAR, a significant increase in root volume was observed in the drought-stressed treatment compared to the control. The total root volume of the GLA8 line significantly exceeds the other two genotypes under drought stress conditions. Its root volume was 48.9% and 73.8% higher than the total root volume of the KAR and Mv9, respectively. Because of the much higher root volume under drought stress conditions, this genotype may store more nutrients and water in its belowground biomass, which can lead to the GLA8 line's better tolerance to water-limited environments as compared to parental wheat genotypes**.**

## Discussion

Using crop wild relatives to increase genetic variability and adaptability is a promising strategy to develop climate-resilient wheat cultivars (Kapazoglou et al. 2023). Within the tertiary gene pool of wheat *Th. intermedium* and *Th. ponticum* have been identified as suitable gene sources for introgression breeding programs (Li and Wang 2009; Pototskaya et al. 2022). The novel GLA8 line showed significant drought tolerance, which is supported by the current investigation.

A. glael, a *Th. intermedium* × *Th. ponticum* artificial hybrid, has been used in Martonvásár wheat pre-breeding program since the early 2000s. While a 6DS.6J^vs^ Robertsonian translocation with high-yielding potential (Türkösi et al. 2024) and a leaf and yellow rust resistant 4StS.1J^vs^S addition line (Kruppa et al. 2025) have been thoroughly characterized, *Thinopyrum* chromosomes in the wheat genetic background remain challenging to identify. One reason could be that various subgenomes (J^r^, J^vs^ and St from *Th. intermedium*, J and J^s^ from *Th. ponticum*) have likely undergone multiple intergenomic rearrangements through meiotic recombination since the development of A. glael (Tsitsin 1979; Kruppa and Molnár-Láng 2016), as was also noted in other open-pollinated cereal hybrids. Chromosome translocations and multivalent formation during the first metaphase of meiosis, for example, are common in the hybrid of the annual *Secale cereale* and the perennial *S. strictum* (Gruner and Miedaner 2021). The rearranged chromosomes of A. glael are difficult to detect using *in situ* hybridization techniques because the hybridization patterns of DNA repetitive probes have altered in comparison to the parental *Th. intermedium* and *Th. ponticum* karyotypes (Kruppa et al. 2016; Xi et al. 2019; Yu et al. 2022). The present work indicated that the FISH pattern of the 3St chromosome identified as 3St(3D) substitution line has considerable similarities to the 3J^s^ chromosome (*Th. intermedium* PI440043) described by Yu et al. (2022). Additionally, the partial visibility of *Thinopyrum* chromosomes when using GISH makes it more difficult to identify them and to determine their homoeologous relationship with the wheat chromosomes (Türkösi et al. 2024; Kruppa et al. 2025). The GBS read coverage analysis strongly suggested that the introgressed *Thinopyrum* chromosome belongs to the homoeologous group 3 as the highest unique read density was mapped on chromosome 3 J. It should be noted that molecular cytogenetic analyses and GBS read coverage data were not entirely concordant. The former indicated the presence of a chromosome belonging to the St genome, whereas the latter suggested assignment to the J genome. This inconsistency could reflect the extensive recombination and structural rearrangements occurring among the subgenomes of A. glael, which in combination with the pronounced polymorphism observed within accessions of *Th. intermedium* and *Th. ponticum* may account for the observed discrepancies when comparisons are made with the *Th. intermedium* reference genome. In line with this hypothesis, Kruppa et al. (2025) also found that a Robertsonian translocation chromosome from a 44-chromosome genotype included two *Thinopyrum* chromosome arms: a 255-Mb 1J^vs^ chromosome fragment and a 175-Mb 4St chromosome fragment while the 1J^vs^ chromosomal arm in the previously used GISH did not produce any fluorescence signals. The development of chromosome-scale reference genomes for wild relatives of cereals is becoming more accessible due to the decreasing cost of long-read sequencing technology and the routinely utilized chromosome conformation capture (Hi-C) techniques (Abrouk et al. 2023; Li et al. 2024). The availability of the reference genome of *Th. ponticum* is anticipated to allow for a more detailed investigation of the genomic structure of A. glael.

The field experiments showed that the morphological traits of 3St(3D) substitution, particularly the fertility and number of grains per plant, were largely identical to wheat parents. These data demonstrate that the introgression of the 3St chromosome has no deleterious effect on wheat's agronomic properties, and it compensates well for the loss of the 3D wheat chromosome, showing a strong homoeologous relationship of them. Like the previously reported 6DS.6J^vs^ Robertsonian translocation line, the GLA8 line showed a better tillering capacity in comparison to wheat parents (Türkösi et al. 2024), implying the presence of a QTL (perhaps with a weaker effect) responsible for tiller development. Additionally, we found that GLA8 plants were consistently 20 cm shorter than both wheat parents, with an average height of 60–65 cm. Among the several wheat/A. glael introgression lines developed during the Martonvásár pre-breeding program, some lack chromosome pair 3D. All observed lines, including those missing the 3D chromosome pair, exhibited either a normal or tall plant height phenotype. Therefore, the semidwarf morphology of GLA 8 cannot be attributed to the absence of chromosome 3D. Our experiments using allele-specific primers confirmed that wheat parents Mv9 and KAR carry *Rht8* (GA-sensitive) and *Rht2* (GA-insensitive) dwarfing alleles, respectively, while the GLA8 line carry both of them (*Rht2* in homozygous and *Rht8* in heterozygous form). Thus, the semidwarf habit of GLA8 resulted from the additive effect of the two dwarfing alleles. Prior research by Rebetzke et al. (2012) with a particular focus on Australian wheat cultivars also assessed the additive effect of the *Rht8* dwarfing allele when combined with the *Rht1* or *Rht2* alleles. On the other hand, *Rht8* is generally classified as a GA-sensitive dwarfing allele, but the magnitude of its GA response and the visible dwarfing it produces depend strongly on genetic background and on interactions with other Rht loci. Ellis et al. (2004) found no difference in leaf elongation rate or sensitivity to GA in the selected lines from the Vigour18 (tall) x Chuan-Mai (*Rht8*) doubled haploid population. This suggests that under certain conditions, the presence of *Rht8* does not affect the response to GA. This is consistent with Tang et al. (2009), who showed that only 39% of the 73 wheat genotypes carrying the *Rht8* allele were sensitive to GA_3_ treatment. In contrast, cultivars that carried *Rht8* together with other *Rht* alleles (*Rht-B1b* or *Rht-D1b*) were mostly GA_3_ insensitive (~ 80%).

The flowering stage has been considered the most drought-sensitive phenological phase as the abortion of male and female gametes directly decreases the yield components, including fertility and grain number per spike (Barnabás et al. 2008; Onyemaobi et al. 2017). These findings were corroborated by our research, as 10 days water withholding during flowering caused a significant decrease in grain yield, which was less severe in GLA8 relative to wheat genotypes, indicating its better tolerance to drought. We found that GLA8 and the wheat parents, particularly Mv9, reacted differently to the stress. For GLA8, the reduction in yield was mostly caused by fewer generative shoots and spikes, and to a lesser degree, by fewer spikelets per spike. In wheat, decreasing the reproductive tillers was not so dominant, but an increase of sterile spikelets as well as a decrease in the number of seeds per spike was more typical phenomenon suggesting a more pronounced malfunction of the reproductive organs. In case of Mv9, the strong drought-induced decrease in grain number was compensated by intensive filling of the remaining grains, which was manifested in the increase of TGW together with higher grain width and grain length. However, these processes were reduced in the GLA8 and KAR genotypes. These results suggest that the wheat line Mv9 tried to compensate for the loss of function of reproductive organs by maintaining the transport of assimilates to the remaining grains.

The smaller decrease in grain yield can be explained by a more efficient water preservation of GLA8, a drought avoidance mechanism observed earlier in tolerant cereal genotypes (Cheng et al. 2016; Pantha et al. 2024). The higher water content of the flag leaves was accompanied by the decreased leaf temperature of GLA8, suggesting more open stomata, a prerequisite to maintain a balanced photosynthetic CO_2_ assimilation, minimizing the water loss via increasing water use efficiency (Arve et al. 2011). A maintained photosynthetic activity of GLA8 was also supported by the less intense chlorophyll degradation evidenced by the color segmentation data, and by smaller drop in values of NDVI, widely used to quantify the density and health of wheat biomass (Lopes and Reynolds 2012), and that of the mND705, related more specifically to chlorophyll content (Sims and Gamon 2002). These data are in line with those of Zhang et al. (2019), who found positive correlation between the NDVI and grain yield in wheat under drought stress applied at the flowering and grain-filling stages. This „Stay-green” (SG) trait allows crop plants to maintain green leaves and photosynthetic capacity for longer periods following anthesis, and to transfer more assimilates into the grains during drought and heat stress conditions (Kamal et al. 2019). In this regard, the GLA8 substitution line performed better than the wheat parents in terms of greenness and reduced leaf senescence, as evidenced by its improved grain yield parameters. It can be concluded that one or more QTLs connected to the SG phenotype can be found on the 3St chromosome. As previously shown for the chromosomes of barley, *Aegilops,* and *Agropyron* introgressed into wheat (Rey et al. 2018; Tiwari et al. 2015; Zwyrtková et al. 2022), flow sorting and sequencing of chromosome 3St from GLA8 may aid in the future exploration of the gene content of this wheatgrass chromosome and the identification of QTLs associated with the SG phenomenon.

The GLA8 substitution line's higher water content and preserved physiological functions suggest changes in cellular systems involved in adaptation to the lower water content. Our gene expression profiling data generally indicates that the most prominent part of the cellular adaptation to drought stress was the stabilization of the membranes and proteins to maintain its proper functions. This was indicated by the highly upregulated level of *DHN3*, which plays a protective role during dehydration (Lopez et al. 2003) and to a lesser extent by *HSP70*, which aids resolubilization of denatured protein aggregates and folding of nascent polypeptides (Xue et al. 2014). The other important goal of the adaptation process is maintaining the cell water status. *PIP2* has been reported to promote water and CO_2_ transfer across the plasma membrane (Wang et al. 2016), hence the significant downregulation of *PIP2* expression in our drought-stressed plants may indicate the active preservation of cellular water by reducing the water transport from the cell. The transfer of water and glycerol from the vacuole to the cytoplasm is coordinated by *TIP3*. The overexpression of *TIP3* suggests that water transfer (together with small osmolites) from the vacuole may also play a role in restoring the disturbed water potential of the cytoplasm (Madrid-Espinoza et al. 2018). It has been previously assessed that the phenylpropanoid pathway protects against excessive reactive oxygen species (ROS) produced under drought stress, which lowers peroxidation and cellular membrane instability and prevents damage to essential macromolecules (nucleic acids, proteins, carbohydrates, and lipids) (Sheteiwy et al. 2019; Sharma et al. 2019). The expression level of *4CL*, one of the key enzymes in phenylpropanoid biosynthesis (Sharma et al. 2019), showed that polyphenol-dependent ROS scavenging was moderately active in the wheat genotypes. It should be mentioned that the general changes in the mentioned mechanisms were the most pronounced in the wheat parental lines, especially in Mv9, while the genotype GLA8 showed the least changes or even an undisturbed status (i.e. *HSP70*, *CL4*). However, a more detailed transcriptome sequencing of GLA8 will be needed to explore the metabolic background of its drought tolerance.

The preserved leaf water status implies that, in comparison to wheat genotypes, the GLA8 root system may react differently to drought stress. The results showed that the GLA8 genotype exhibited a significant increase in root diameter and a moderate increase in root volume, in comparison with wheat genotypes. In our opinion, increasing root diameter and volume results in a reduced surface-to-volume ratio, which leads to better water storage in case of water scarcity. This finding on GLA8 is consistent with those of previous studies (Christmann et al. 2013, Zhang et al. 2025), which outlined how increasing root biomass-including length, density, and volume, is a crucial strategy to increase crops' resistance to drought and lessen the impact of stress on plant growth and yield loss.

In summary, the current study confirmed that bread wheat's tolerance to drought may be enhanced by beneficial genes found in wild relatives adapted to harsh environments (Nevo and Chen 2010; Pour-Aboughadareh et al. 2021). Wheatgrass (*Thinopyrum*) species have been frequently used as gene sources in a variety of breeding programs (Wang 2011; Ceoloni et al. 2013). In addition to other advantageous agronomic characteristics, it has been demonstrated that accessions from *Th. intermedium* and *Th. ponticum* are drought-tolerant. Using state-of-the-art GBS methods, the identification of wheatgrass chromosomes was significantly facilitated in addition to cytogenetic approaches. However, the complexity and presumably the highly rearranged genome structure of A. glael remain a considerable barrier to identifying introgressed chromosomes. Field trials confirmed that 3St chromosomes compensated well for the loss of wheat chromosome 3D. Moreover, the wheatgrass chromosome caused improved tolerance against drought stress during the flowering stage. This tolerance mechanism includes the maintenance of water content, cellular functions, and functions of reproductive organs manifested in higher grain yield relative to wheat parents. However, further chromosome engineering efforts will be required to decrease the size of the alien introgressed chromosome and delineate the QTL region responsible for drought tolerance.

## Supplementary Information

Below is the link to the electronic supplementary material.

Supplementary file1 (DOCX 445 kb)

## Data Availability

*Thinopyrum intermedium* sequence data were produced by the US Department of Energy Joint Genome Institute, available at: http://phytozome.jgi.doe.gov/. Genotyping-by-sequencing datasets generated for this study is available at NCBI SRA: SRR33094015 under BioProject: PRJNA1248497; NCBI SRA: SRR30734337, SRR30734336, and SRR30734335 under BioProject: PRJNA1162751. Seeds of the GLA8 substitution line are freely available for non-commercial purposes upon the acceptance of the provider’s Material Transfer Agreement. Email: molnar.istvan@atk.hun-ren.hu.
