# Supplementary material for: Replacement of chromosome 3D with Thinopyrum chromosome 3St led to increased drought tolerance during the flowering stage in wheat
Source: Plant Cell Rep. 2025 Oct 18;44(11):242. doi: 10.1007/s00299-025-03632-5 (PMC12535497; doi:10.1007/s00299-025-03632-5)
Supplement: Supplementary file 1 — Supplementary file1 (DOCX 445 kb) [file 299_2025_3632_MOESM1_ESM.docx]

**Supplementary table 1** Genotype main effects on agronomic traits

| **Trait** | **F** | **p** | **Significance** |
| --- | --- | --- | --- |
| Plant height | 201.38 | <0.001 | significant |
| Spikes per plant | 9.22 | <0.001 | significant |
| Spike length | 61.10 | <0.001 | significant |
| Spikelet per main spike | 0.44 | 0.644 | non- significant |
| Seed per main spike | 8.10 | 0.001 | significant |
| Fertility (grains per spikelet) | 8.29 | 0.001 | significant |
| Yield | 1.43 | 0.245 | non- significant |
| Culm length | 169.96 | <0.001 | significant |

**Supplementary table 2** Environment main effects on agronomic traits

| **Trait** | **F** | **p** | **Significance** |
| --- | --- | --- | --- |
| Plant height | 10.89 | <0.001 | significant |
| Spikes per plant | 12.77 | <0.001 | significant |
| Spike length | 17.47 | <0.001 | significant |
| Spikelet per main spike | 20.31 | <0.001 | significant |
| Seed per main spike | 1.21 | 0.304 | non- significant |
| Fertility (grains per spikelet) | 0.15 | 0.858 | non- significant |
| Yield | 13.78 | <0.001 | significant |
| Culm length | 16.04 | <0.001 | significant |

**Supplementary table 3** Genotype **×** Environment main effects on agronomic traits

| **Trait** | **F** | **p** | **Significance** |
| --- | --- | --- | --- |
| Plant height | 1.39 | 0.243 | non- significant |
| Spikes per plant | 3.34 | 0.014 | significant |
| Spike length | 1.06 | 0.381 | non- significant |
| Spikelet per main spike | 2.42 | 0.055 | marginally significant |
| Seed per main spike | 0.78 | 0.543 | non- significant |
| Fertility (grains per spikelet) | 1.06 | 0.384 | non- significant |
| Yield | 2.39 | 0.058 | marginally significant |
| Culm length | 1.91 | 0.117 | non- significant |

**Supplementary Table 4** Details of markers used for gene expression analysis

| Name | Primer Sequence | Efficiency | | | Target ID |
| --- | --- | --- | --- | --- | --- |
|  |  | GLA8 | KAR | Mv9 |  |
| GAPDH-255-Fmod | CCTCCAGATTTCCTTCCCCA | 2.014 | 2.014 | 2.039 | XM_044563950.1 |
| GAPDH-604-R | CAGCCTTGTCCTTGTCAGTG |  |  |  |  |
| EF1-890-F | GCAGACTCACATCAACATCG | 2.019 | 2.129 | 2.022 | XM_044543456.1 |
| EF1-1015-R | CTTCCTTCTCAAACCTCTCG |  |  |  |  |
| Glael-DHN3-97F | TTACACAACACAGCCACGAG | 1.891 | 1.906 | 1.870 | XM_044561020.1 |
| DHN3-408Rmod | CACGCCATCATCCTCAGAC |  |  |  |  |
| HSP70-1445-F | CCATCCCCACCAAGAAGG | 2.093 | 2.079 | 2.079 | AF005993.1 |
| HSP70-1543-R | TTGCCCAGGAGGTTGTTGT |  |  |  |  |
| PIP2-342-Fmod | GCCCAAGGTGTCGCTGCTA | 2.013 | 1.945 | 1.960 | KU366816.1 |
| PIP2-553-R | CGGAGAAGACGGTGTAGACG |  |  |  |  |
| TIP3-608-Fmod | TCTGGAGGCGGTGATGAC | 1.949 | 1.916 | 1.940 | AB535600.1 |
| TIP3-741-Rmod | CTGCGAGTATGTTAGCCC |  |  |  |  |
| 4CL-1697-Fmod | GACGCTGGTGAGATCCCG | 2.017 | 1.944 | 1.946 | XM_044476428.1 |
| 4CL-1866-Rmod | CTTCCCTGAGTTGCCTCC |  |  |  |  |

**Supplementary Fig. 1**


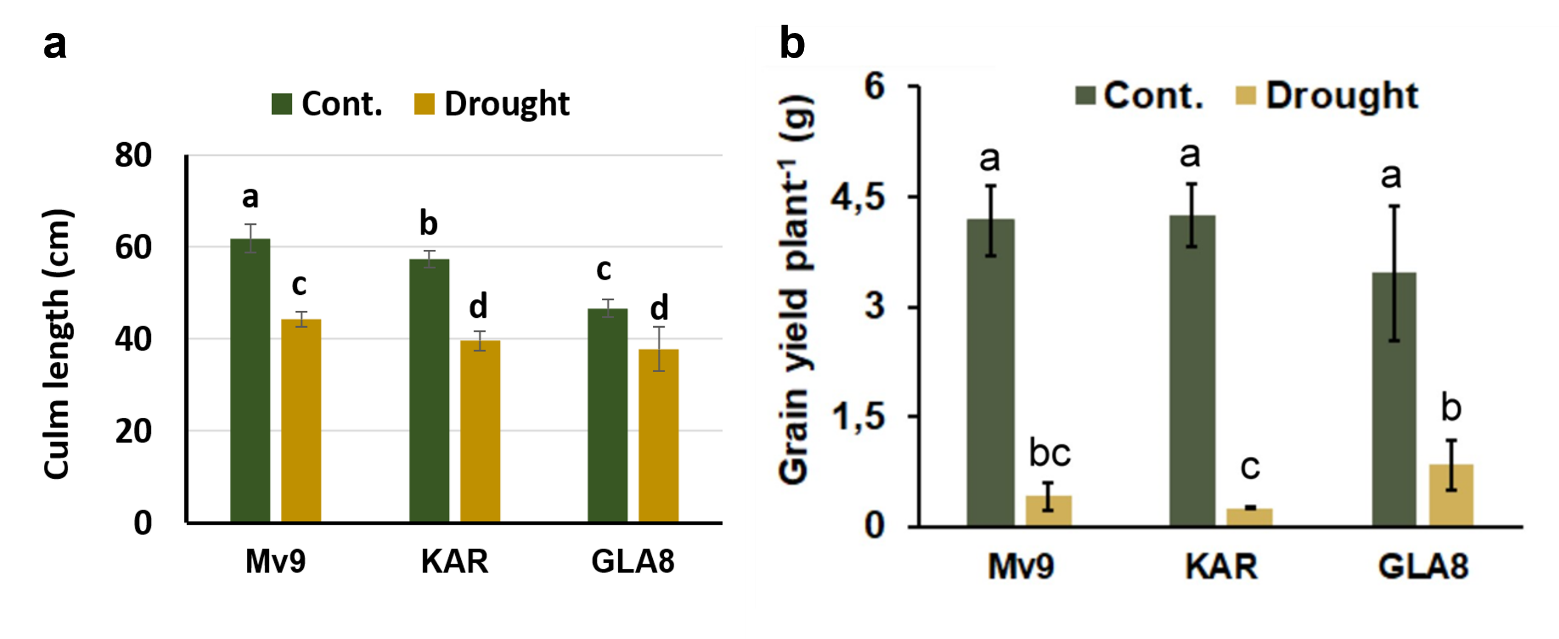


Culm length (a) and grain yield per plant (b) in drought tolerance tests for Mv9 and KAR wheat parents, and the GLA8 substitution line. Standard errors of the mean are shown as error bars. Two-way ANOVA with Tukey’s post hoc tests were performed for each genotype under both control and drought stress treatments (α = 0.05, n = 8). Different letters above the error bars indicate statistically significant differences between treatments (Cont.=Control)
